# Spatial patterns of phylogenetic diversity and endemism in the Western Ghats, India: A case study using ancient predatory arthropods

**DOI:** 10.1002/ece3.8119

**Published:** 2021-11-12

**Authors:** D. K. Bharti, Gregory D. Edgecombe, K. Praveen Karanth, Jahnavi Joshi

**Affiliations:** ^1^ CSIR‐Centre for Cellular and Molecular Biology Uppal Road Hyderabad India; ^2^ Natural History Museum London UK; ^3^ Centre for Ecological Sciences Indian Institute of Science Bangalore India

**Keywords:** biodiversity hotspots, centipedes, diversity gradients and endemism, peninsular India, species richness

## Abstract

The Western Ghats (WG) mountain chain in peninsular India is a global biodiversity hotspot, one in which patterns of phylogenetic diversity and endemism remain to be documented across taxa. We used a well‐characterized community of ancient soil predatory arthropods from the WG to understand diversity gradients, identify hotspots of endemism and conservation importance, and highlight poorly studied areas with unique biodiversity. We compiled an occurrence dataset for 19 species of scolopendrid centipedes, which was used to predict areas of habitat suitability using bioclimatic and geomorphological variables in Maxent. We used predicted distributions and a time‐calibrated species phylogeny to calculate taxonomic and phylogenetic indices of diversity, endemism, and turnover. We observed a decreasing latitudinal gradient in taxonomic and phylogenetic diversity in the WG, which supports expectations from the latitudinal diversity gradient. The southern WG had the highest phylogenetic diversity and endemism, and was represented by lineages with long branch lengths as observed from relative phylogenetic diversity/endemism. These results indicate the persistence of lineages over evolutionary time in the southern WG and are consistent with predictions from the southern WG refuge hypothesis. The northern WG, despite having low phylogenetic diversity, had high values of phylogenetic endemism represented by distinct lineages as inferred from relative phylogenetic endemism. The distinct endemic lineages in this subregion might be adapted to life in lateritic plateaus characterized by poor soil conditions and high seasonality. Sites across an important biogeographic break, the Palghat Gap, broadly grouped separately in comparisons of species turnover along the WG. The southern WG and Nilgiris, adjoining the Palghat Gap, harbor unique centipede communities, where the causal role of climate or dispersal barriers in shaping diversity remains to be investigated. Our results highlight the need to use phylogeny and distribution data while assessing diversity and endemism patterns in the WG.

## Graphical abstract

Soil arthropod communities play an important role in the ecosystem, but have been poorly studied in terms of large‐scale patterns of diversity and distribution from a phylogenetic perspective. We studied centipedes, a group of ancient predatory soil arthropods, across the climatic gradient of the Western Ghats, India, a global biodiversity hotspot with a complex geological history, using extensive primary distribution data and a robust phylogeny. Our results reveal a latitudinal gradient in phylogenetic diversity of centipedes along the mountain range, identify unique hotspots of phylogenetic endemism, provide support to the existence of a past rainforest refuge, and indicate structuring of centipede communities across various subregions.

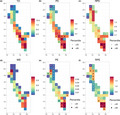

## 1 INTRODUCTION

**FIGURE 1:**
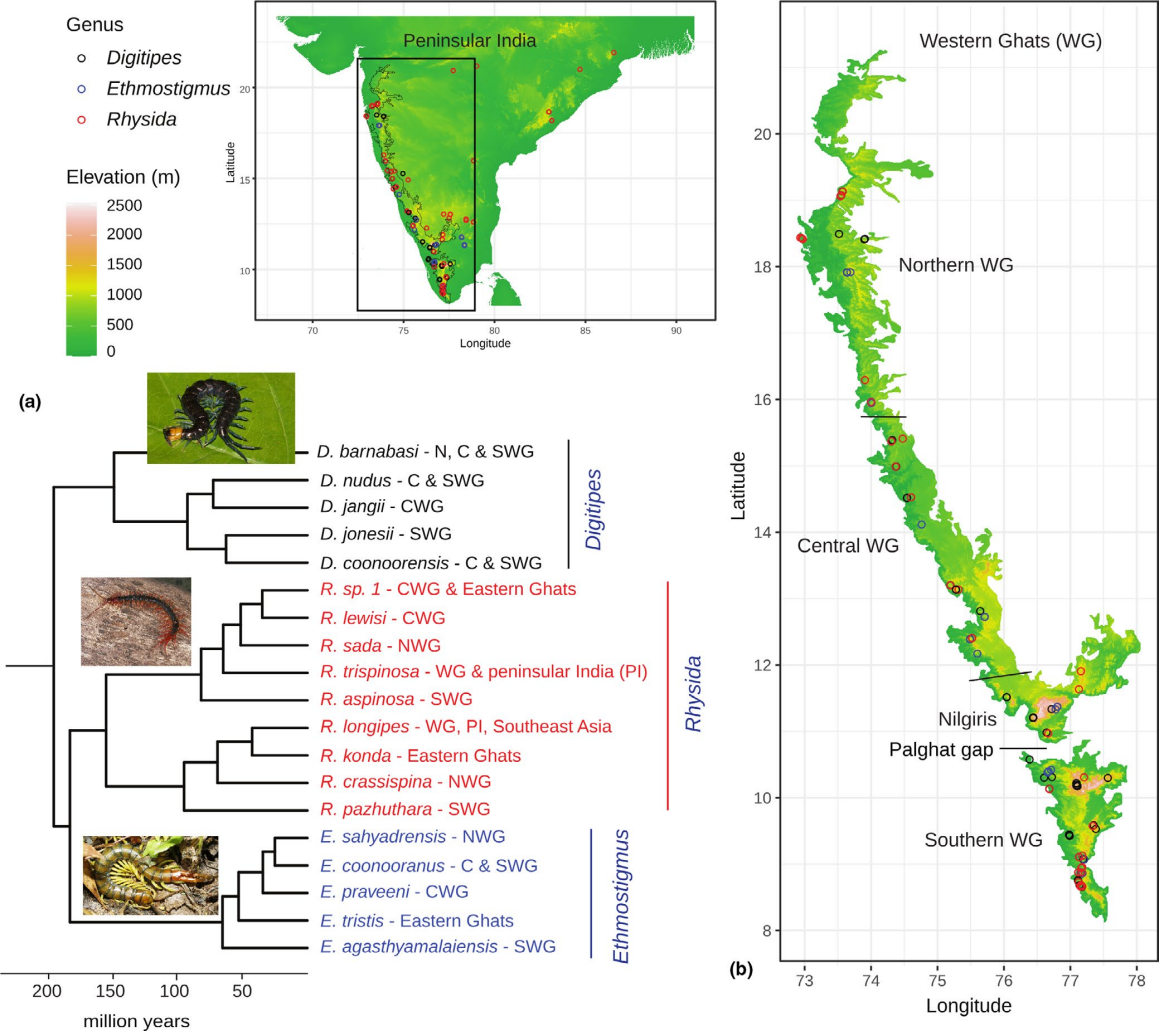
(a) Bayesian phylogeny for Western Ghats Otostigminae (Joshi et al., 2020) (b) A detailed map of the distribution records across peninsular India with a focus on scolopendrid centipede communities of the Western Ghats

The Western Ghats (WG) is a 1600‐km (8°N – 21°N) long mountain chain that runs along the west coast of peninsular India. It has been identified as a global biodiversity hotspot due to its high diversity and endemicity (Myers et al., 2000). A recent biodiversity assessment reported that 30% of India's biodiversity is found in the WG, with a high proportion of endemic species (CEPF, 2016). This mountain chain has been divided into four phytogeographic subregions corresponding to northern WG (river Tapi to Goa), central WG (river Kali to Coorg), Nilgiris, and the southern WG (Anamalai, Palani, and Cardamom hills) (Subramanyam & Nayar, 1974; Figure 1). The WG mountain chain has a prominent 30‐km‐wide break at 11°N, known as the Palghat Gap (Figure 1), which separates the Nilgiris from the southern WG and is thought to be an important biogeographic barrier (Joshi & Karanth, 2013; Robin et al., 2015; Vijayakumar et al., 2016). Biogeographically, the WG has a complex history as it was part of the Gondwanan supercontinent ca. 200 Ma ago and merged with Asia only recently, ca. 50 Ma (Joshi & Karanth, 2011 and references therein). As a result, the WG harbors taxa with both Gondwanan and Asian affinities as well as many endemic radiations (Bossuyt & Milinkovitch, 2001; Gower et al., 2011; Joshi & Edgecombe, 2019; Joshi & Karanth, 2011; Surveswaran et al., 2020).

The southern parts of the WG remained climatically stable during the massive Cretaceous (ca. 65 Ma) volcanic activity, which led to widespread extinctions in peninsular India. This subregion of the WG is hypothesized to have acted as a refugium for wet evergreen species (SWG refuge hypothesis—Joshi & Karanth, 2013 and references therein), with predictions of older plant and animal lineages in the southern WG and relatively younger and phylogenetically nested lineages in the central and the northern WG. In addition to these geoclimatic processes, there is also a contemporary seasonality gradient along the WG, with northern latitudes showing greater temperature and precipitation seasonality than the southern latitudes (Bose et al., 2019; Joshi & Karanth, 2013; Page & Shanker, 2020). Studies based on distributions of plant and animal species from this region have revealed a decreasing trend in diversity from the southern to the northern WG (plants: Davidar et al., 2005, Page & Shanker, 2020; snails: Aravind et al., 2005; frogs: Daniels, 1992, Aravind & Gururaja, 2011). The southern WG has also been associated with high endemism (Aravind & Gururaja, 2011; Daniels, 1992; Divya et al., 2020). However, these patterns are based on species distributions and have not been investigated using explicit phylogenetic data, with the exception of recent studies on the diversity of woody plants (Bose et al., 2019; Divya et al., 2020).

While taxonomic diversity (TD) is one of the most commonly used measures to characterize a community, it treats all species as independent units, which may not be true. To address this, phylogenetic diversity (PD) was proposed to explicitly incorporate the evolutionary history for each species, which would reflect the accumulated evolutionary history of a community (Faith, 1992). Further, the geographic distribution of a species along with phylogenetic divergence can be incorporated for all the taxa in a given region through phylogenetic endemism (PE), a metric that weights the branch lengths of each lineage by its respective geographic range (Rosauer et al., 2009). The use of PD and PE allows us to assess the roles of ecological, historical, and evolutionary processes that structure communities, and their usefulness has been demonstrated in multiple biodiverse and complex landscapes (Azevedo et al., 2020; Fenker et al., 2020; Mishler et al., 2014), but remains limited in Asian tropical forests (Bose et al., 2019; Divya et al., 2020; Tamma & Ramakrishnan, 2015). In such regions, including the WG, it is important to document the patterns of both phylogenetic diversity and endemism to understand the ecological and evolutionary processes shaping biodiversity and to identify areas of conservation importance.

While most existing global macroecological studies focus on plants (Massante et al., 2019), birds (Jetz et al., 2014), mammals (Safi et al., 2011), and herpetofauna (Fritz & Rahbek, 2012), arthropods have been largely ignored (Beck & McCain, 2020). Among arthropods, predatory soil‐dwelling communities have been particularly neglected in macroecological studies (Finch et al., 2008), as they typically consist of many cryptic species occurring in low abundance, making them difficult to detect. This is coupled with a lack of taxonomic expertise in identifying them to the species‐level, as well as the presence of many undescribed species/lineages. In many ecosystems, predatory soil arthropods are likely to be the oldest lineages and play an important role in maintaining the ecosystem, but their diversity patterns remain poorly understood.

Centipedes (class: Chilopoda) are one such group of soil arthropods, which represent one of the four main myriapod lineages, with a 420‐million‐year(Ma)‐old fossil history, making them some of the oldest living terrestrial predators (Edgecombe & Giribet, 2019). The centipede family Scolopendridae from the WG offers a unique opportunity for conducting macroecological studies as it is well‐characterized taxonomically and phylogenetically. It is among the oldest (Late Cretaceous—100 Ma) soil arthropod communities in the WG and is one of the most diverse centipede groups in tropical Asian forests. Globally, the family Scolopendridae has more than 400 species, of which ~35 species belonging to seven genera occur in the WG.

The WG scolopendrid community has been extensively studied using both morphology and molecular data, resulting in the discovery of many endemic species and radiations (Joshi & Edgecombe, 2018; Joshi et al., 2020). These studies are accompanied by detailed primary distribution data spanning large latitudinal (8°N – 20°N) and elevational gradients (100 – 2,400 meters from mean sea level (msl)). It is noteworthy that molecular data were integrated with morphology and species ecology in these studies, as the latter often receive lesser attention in the current barcoding era (Padial et al., 2010). The scolopendrid centipedes of the WG vary in their endemicity patterns, ranging from narrow‐range endemics to species with wide distributions across the WG or exceptionally widespread distribution into SE Asia.

In this study, we focus on a diverse and predominantly tropical subfamily, Otostigminae (Figure 1). Among the three genera within Otostigminae studied here, *Digitipes* and *Ethmostigmus* occur in wet forests, where *Digitipes* is largely restricted to the WG, while *Ethmostigmus* has dispersed to the wet forests of the Eastern Ghats in the past (Joshi & Edgecombe, 2019). In contrast, *Rhysida* is widely distributed across various habitat types throughout peninsular India. In terms of biogeographic history, there are instances of out of India dispersals across genera, but the origin of most species is in the Indian subcontinent/WG. There is evidence for dispersals and range expansions from the southern WG in *Digitipes* (Joshi & Karanth, 2013), a likely origin of *Ethmostigmus* species in south/central WG (Joshi & Edgecombe, 2019), and out of India dispersals in *Rhysida* (Joshi et al., 2020). The evidence from these larger phylogenies indicates that the WG species evolved in situ rather than through dispersals from NE India or SE Asia. We used this group of centipedes for assessing hypotheses related to the patterns of diversity and endemism for predatory soil arthropods in tropical forests at a community level. Globally, such datasets are rare, most of them consisting of island communities (Caribbean: Crews & Esposito, 2020; Azores: Borges & Hortal, 2009; Galapagos: Peck, 2006; Hawaii: Gillespie, 2002).

Our aim in this study is to understand how the diversity of the centipede community is structured in the WG. Based on distribution and biogeographic studies of plants and animals from this region (plants: Davidar et al., 2005, Page & Shanker, 2020; snails: Aravind et al., 2005; frogs: Aravind & Gururaja, 2011, Daniels, 1992), we expect a latitudinal diversity gradient (LDG) in which diversity increases from higher to lower latitudes in the WG. Additionally, the SWG refuge hypothesis provides expectations of ancient and high diversity in the southern WG and Nilgiris as a result of climatic stability allowing more time for speciation, while central and northern WG taxa have fewer and relatively younger (<65 Ma) lineages. These two hypotheses are not mutually exclusive and provide similar expectations, where the causal process for the LDG is generally associated with climatic stability (among other drivers), while the SWG refuge hypothesis specifically originates from geoclimatic processes related to Cretaceous volcanism in this landscape. To explore these questions, we used species distribution models to map the spatial patterns of diversity and endemism instead of relying only on point locations from sampling surveys. We then used predictions from these models to compare patterns of taxonomic diversity and endemism with phylogenetic diversity and endemism to identify areas with unique diversity. We asked the following specific questions:

1. How is centipede diversity distributed in the WG?
  Based on the LDG and SWG refuge hypotheses, we expect to see a decreasing gradient in diversity from southern to northern WG.
2. Are there hotspots within the hotspot?
  Since diversity may not be uniformly distributed given the climatic and topographic heterogeneity in the WG, we examined whether there are areas represented by disproportionately high diversity and endemism within the WG biodiversity hotspot.
3. What are the patterns of species turnover across the WG?
  We assessed whether taxonomic and phylogenetic compositions are unique to each of the biogeographic subregions in the WG.

## 2 METHODS

### 2.1 Species distribution models

Primary location data (*n* = 100) for 19 species in the three genera of the centipede family Scolopendridae (Subfamily Otostigminae): *Digitipes* Attems, 1930 (five species)*, Ethmostigmus* Pocock, 1898 (five species), and *Rhysida* Wood, 1862 (nine species) were obtained by systematic sampling across the Western Ghats (WG) from 2008 to 2010, spanning its latitudinal and elevational gradients (Joshi & Edgecombe, 2013, 2018; Joshi & Karanth, 2012; Joshi et al., 2020) (see Appendix S1). These data were supplemented with opportunistic sampling which continued to 2018. These locations spanned the extent of peninsular India, with a focus on the wet forests of the WG (Figure 1b). Since it is challenging to identify centipede species in the field, specimens associated with the primary location data were collected and identified in the laboratory based on microscopic examination of morphological characters. Species identity was also assessed through molecular phylogenetic and species delimitation analyses (Joshi & Edgecombe, 2013, 2018; Joshi & Karanth, 2012; Joshi et al., 2020). State forest department permits were obtained to collect centipedes in protected areas, and specimens were preserved in 70% ethanol. A few secondary locations (*n* = 10) were obtained from published sources, where we were certain about the species identification based on morphological characters described in the source literature (Jangi & Dass, 1984).

It is difficult to determine true absence for a group such as centipedes due to their low abundance, morphologically cryptic nature, and lack of systematic information on their distribution in less explored areas such as the WG. Therefore, we chose to model species distributions using Maxent version 3.4.1 (Phillips et al., 2006), which uses a presence‐background approach to predict species distributions that have been shown to perform well across species, regions (Elith et al., 2006; Phillips & Dudík, 2008), and a range of sample sizes (Hernandez et al., 2006; Wisz et al., 2008). Maxent compares the environment at presence locations against background locations drawn from the model extent to arrive at a model of relative suitability for a species based on the underlying environmental variables (Elith et al., 2011; Merow et al., 2013). We ran Maxent models for each species for the model extent of peninsular India (8° ‐ 24°N, 68° ‐ 91°E) at 30 arc second resolution (0.0083 × 0.0083 degree resolution, 0.93 × 0.93 km at equator).

In these models, we used presence locations from each species (mean = 9.9 presence locations per species, range = 3–42 presence locations per species) and 10,000 background locations selected probabilistically from a bias layer. The bias layer was derived from a model built using a pooled dataset of presence locations including all species, 19 environmental variables and elevation from WorldClim (Fick & Hijmans, 2017) and a soil type layer (ATREE Spatial Archive, 2020) as predictor variables, 10,000 random background locations, and default Maxent parameters. Higher values of habitat suitability predictions (ranging from 0 to 1) obtained from this model represent areas with environments which are more likely to have been sampled, thus representing sampling bias across the model extent (Phillips et al., 2009).

The predictor variables for species‐specific Maxent models consisted of six primary environmental layers and elevation from the WorldClim database (Fick & Hijmans, 2017) and a soil type layer (ATREE Spatial Archive, 2020). This reduced subset of WorldClim environmental variables (annual mean temperature, maximum temperature of the warmest month, minimum temperature of the coldest month, annual precipitation, precipitation of the wettest month, and precipitation of the driest month) has been recommended for species lacking ecological information or in community‐level studies involving models of several species (Low et al., 2021). The use of these primary variables reduces complexity in the predictor dataset which can lead to model overfitting (Zeng et al., 2016) and avoids the inclusion of several correlated composite variables, while still allowing the use of different kinds of predictors (Low et al., 2021). For each species, we built separate models using six different combinations of predictor transformations known as feature classes (LQH, LQ, QH, L, Q, H, where L—linear, Q—quadratic, H—hinge), each of which was tuned using ten different regularization parameters (0.5 to 5 with intervals of 0.5; Maxent default is 1), which smoothens model predictions (Elith et al., 2011). This was done as Maxent defaults for choosing feature classes and regularization parameters might not be appropriate across all species, and species‐specific model fitting and tuning are recommended to build simpler models with better transferability (Hallgren et al., 2019; Low et al., 2021; Radosavljevic & Anderson, 2014).

To select the combination of feature classes and regularization multipliers in the best performing models, we carried out model evaluation using a cross‐validation approach. Test and training datasets were obtained using four masked geographically structured partitions (Radosavljevic & Anderson, 2014) for species with 20 or more presence locations and k‐1 jackknifing (Shcheglovitova & Anderson, 2013) for species with fewer presence locations. Average evaluation metrics calculated across partitions included measures of model transferability – OR_MTP_ (omission rate of test presences in model predictions using a threshold of minimum training presence) and AUC_DIFF_ (difference between training and test Area Under the Receiver Operator Curve, which assesses model overfitting), and model discriminatory ability – AUC_TEST_ (interpreted as the probability that the model ranks a randomly picked presence location higher in habitat suitability than a randomly picked background location, Fielding & Bell, 1997; Low et al., 2021). For each species, models with AUC_TEST_>0.6 were compared sequentially to select those with minimum OR_MTP_, followed by minimum AUC_DIFF_ and finally maximum AUC_TEST_. For each species, the best performing model was then used to obtain predictions of habitat suitability across the model extent of peninsular India.

We additionally evaluated two other predictor datasets—1. all WorldClim variables + soil type (21 predictors in total) and 2. ecologically relevant WorldClim variables + soil type (7 predictors in total), along with an alternate AICc‐based model selection procedure, details of which are provided in Appendix S2.

Spatial data were processed using the packages “rgeos” (Bivand & Rundel, 2017), “raster” (Hijmans, 2020), and “sp” (Bivand et al., 2013; Pebesma & Bivand, 2005), and Maxent models were run using the package “ENMeval” (Muscarella et al., 2014) and “dismo” (Hijmans et al., 2017) in R 3.6.1 (R Core Team, 2019).

### 2.2 Diversity and endemism measures

The diversity and endemism measures derived from predicted distributions at the scale of peninsular India were cropped for the WG for further analysis, since this biodiversity hotspot is the focus of our study where systematic sampling was undertaken. We aggregated continuous Maxent predictions of relative habitat suitability to obtain maps at a scale of 0.83 × 0.83 degrees (93 × 93 km at equator). We assigned the maximum value of habitat suitability among the underlying cells to the larger aggregated cell and applied a threshold of maximum sum of sensitivity and specificity (Liu et al., 2005) to convert it into a presence–absence map for each species.

We used these binary maps to calculate taxonomic diversity (TD) and weighted endemism (WE) along with their phylogenetically informative counterparts—phylogenetic diversity (PD) and phylogenetic endemism (PE) for each cell in the model extent. As compared to taxonomic indices of diversity and endemism, the phylogenetic indices provide additional information on evolutionary relationships between species present within a community, helping to distinguish between closely and distantly related species. The diversity indices enable us to test the latitudinal diversity gradient hypothesis in the WG, where we expect to observe increasing diversity with decreasing latitude. Combined with the endemism indices, they help to test predictions from the southern WG refuge hypothesis from which we would expect to see high diversity and endemism within the southern WG. Additionally, both the indices of endemism allow us to identify hotspots consisting of range‐restricted species within the WG, while PE additionally identifies evolutionarily unique and range‐restricted species.

TD is calculated by stacking species distributions and summing species presences for each cell, whereas WE is calculated by scaling species presence with its range size (number of cells in the predicted map in which a species is present) and summing this across species found in a cell (Crisp et al., 2001). A species time tree for the subfamily Otostigminae based on a combined dataset of mitochondrial and nuclear markers (Joshi et al., 2020) was used to calculate PD and PE. PD is calculated by summing up branch lengths in the minimum spanning path derived from the larger phylogenetic tree, which includes all the species present in a cell (Faith, 1992). PE additionally scales each branch length in the minimum spanning path with its range size prior to summation across the lineages in a cell (Rosauer et al., 2009). Both PD and PE are presented as proportion of total tree length (range: 0 – 1) (Table 1).

**TABLE 1:** Summary of diversity, endemism, and turnover indices used in this study

S. No	Abbreviation	Index	Definition	Inference
1	TD	Taxonomic Diversity	The total count of species present within a grid cell. (range: 0‐total species count)	Measure of diversity considering species as independent units, where larger values indicate greater species counts.
2	PD	Phylogenetic Diversity (Faith, 1992)	The sum of branch lengths of a minimum spanning path linking species present within a grid cell to the root of the tree. Scaled by total length of phylogenetic tree. (range: 0–1)	Measure of diversity considering evolutionary relationships between species from a phylogenetic tree, where larger values indicate the presence of relatively older lineages.
3	WE	Weighted Endemism (Crisp et al., 2001)	Summation of the inverse of range size over each species present within a grid cell. (range: 0‐total species count)	Measure of endemism, where larger values indicate greater prevalence of range‐restricted species.
4	PE	Phylogenetic Endemism (Rosauer et al., 2009)	Summation of branch lengths weighted by range size, for each branch in a minimum spanning path linking species in a grid cell to the root of the tree. Scaled by total length of phylogenetic tree. (range: 0–1)	Measure of endemism considering the evolutionary relationships between species as inferred from a phylogenetic tree, where larger values indicate the presence of older lineages and/or restricted range.
5	RPD	Relative Phylogenetic Diversity (Mishler et al., 2014)	Ratio of observed phylogenetic diversity over the same index calculated using an identical phylogenetic tree but with equal branch lengths.	Values significantly larger than 1 indicate the overrepresentation of lineages with long branch lengths.
6	RPE	Relative Phylogenetic Endemism (Mishler et al., 2014)	Ratio of observed phylogenetic endemism over the same index calculated using an identical phylogenetic tree but with equal branch lengths.	Values significantly larger than 1 indicate the overrepresentation of lineages with restricted range sizes and long branch lengths.
7		Pairwise Simpson's dissimilarity index (Lennon et al., 2001; Simpson, 1943)	The lower of the species numbers unique to each grid cell divided by sum of this value with the number of common species between grid cells.	Compositional differences between communities explained by species replacement alone, where higher values indicate greater dissimilarity.
8	PhyloSor_Turn_	Phylogenetic Turnover (Leprieur et al., 2012)	The lower of the sums of branch lengths unique to each grid cell divided by the above value added to the sum of branch lengths common between grid cells.	Evolutionary dissimilarity between communities explained by loss of branch lengths not explained by differences in phylogenetic diversity, where higher values indicate greater dissimilarity.

To compare and identify differences in the relative distribution of evolutionary ages among lineages present within different communities, we used relative phylogenetic diversity (RPD) and relative phylogenetic endemism (RPE) (Table 1). These indices compare the observed values of PD and PE with those obtained from a phylogenetic tree with equal branch lengths, which allows us to understand whether there is an over‐representation of evolutionarily old or young lineages within a community. This information can provide insights into the biogeographic history or ecological processes operating in a region, for example, it can help to distinguish between centers of neo‐ and paleo‐endemism (Mishler et al., 2014).

RPD and RPE were calculated as the ratio of PD and PE derived from the actual phylogenetic tree over the same indices derived from a phylogenetic tree where species relationships remain the same but lineages have equal branch lengths. An RPD or RPE value of 1 indicates that the lineages present in a cell have equal branch lengths. Values larger than 1 indicate regions harboring species with longer branch lengths in the phylogenetic tree, and values smaller than 1 indicate regions harboring species with shorter branch lengths in the phylogenetic tree (Mishler et al., 2014).

We generated null distributions of phylogenetic diversity and endemism measures for comparison with observed values by random assignment of species occurrences to grid cells in the model extent without replacement, while keeping the taxonomic diversity and range size of each species constant (Mishler et al., 2014). This procedure randomizes the species identities in a grid cell, and in doing so picks a random set of tip labels from the phylogenetic tree for each grid cell as compared to observed species identities (Mishler et al., 2014). The diversity and endemism indices and their corresponding null distributions were calculated using Biodiverse 3.1 (Laffan et al., 2010).

We used measures of beta diversity to assess whether taxonomic and phylogenetic composition varies between the different biogeographic subregions recognized along the WG. To identify patterns of taxonomic beta diversity, we used the Simpson dissimilarity index, which describes the variation in species composition due to species turnover alone (Baselga, 2010). We also calculated its phylogenetic counterpart in the form of PhyloSor_Turn_, which measures the loss of branch lengths between communities not explained by differences in phylogenetic diversity (Leprieur et al., 2012). The taxonomic and phylogenetic indices of turnover (Table 1) were used for cluster analysis using the UPGMA algorithm (Michener & Sokal, 1957) to group subregions in the WG (we applied cutoffs to obtain *k* = 4 clusters) based on patterns of species composition. Beta diversity and phylogenetic beta diversity indices were calculated using the “betapart” package (Baselga, 2010) in R 3.6.1 (R Core Team, 2019).

## 3 RESULTS

### 3.1 Maxent predictions for the scolopendrid community

For all the Maxent models, at least two‐thirds of the test presence locations were correctly predicted across species (OR_MTP_ range: 0–0.33), with 14 out of 19 species having AUC values greater than 0.8 (AUC_TEST_ range: 0.6002–0.9952 and AUC_DIFF_ range: 0–0.2284) (Table 2). For 15 of 19 species, a regularization parameter greater than 1 was selected, and for 17 species, a simpler subset of feature classes as compared to Maxent defaults was selected in the final model. A qualitative examination of the habitat suitability maps revealed substantial variation in predictions across different predictor datasets and model selection methods for *Rhysida sada*, and high overprediction for *Rhysida* sp. 1. Hence, for these species, available presence locations were used instead of model predictions for diversity and endemism calculations. *Rhysida konda*, an Eastern Ghats endemic, did not have predicted distribution in any of the grid cells within the Western Ghats (WG) and was thus not included in the further analysis. Maxent results showed that variables related to precipitation and temperature extremes were important in predicting the distributions of centipede species (Appendix S2).

**TABLE 2:** Model settings and evaluation metrics of Maxent species distribution models run at 0.0083° × 0.0083° resolution for peninsular India. n—number of occurrence records used in the model, FC—feature class combination, RM—regularization multiplier, OR_MTP_—average omission rate of test locations using a threshold of minimum training presence, AUC_DIFF_—average difference between test and training area under the receiver operator curve (AUC), AUC_TEST_—average test AUC. For species with 20 or more presence locations, averages of evaluation metrics were calculated using four masked geographically structured partitions and k‐1 jackknifing for species with fewer than 20 locations. Presence locations were used instead of Maxent model predictions for species highlighted in gray (see results section)

S. No.	Genus	Species	*n*	FC	RM	OR_MTP_	AUC_DIFF_	AUC_TEST_
1	*Digitipes*	*barnabasi*	23	L	5.0	0.0417	0.0117	0.6118
2	*Digitipes*	*coonoorensis*	14	Q	2.0	0.0714	0.0315	0.9452
3	*Digitipes*	*jangii*	9	Q	4.0	0.1111	0.0602	0.9004
4	*Digitipes*	*jonesii*	27	H	1.0	0.0833	0.0617	0.8967
5	*Digitipes*	*nudus*	6	Q	1.0	0.1667	0.0362	0.9325
6	*Ethmostigmus*	*agasthyamalaiensis*	6	H	5.0	0.1667	0.0302	0.9483
7	*Ethmostigmus*	*coonooranus*	6	L	3.0	0.1667	0.0484	0.8063
8	*Ethmostigmus*	*praveeni*	4	LQ	0.5	0.2500	0.0199	0.9726
9	*Ethmostigmus*	*sahyadrensis*	5	LQH	0.5	0.2000	0.0041	0.9930
10	*Ethmostigmus*	*tristis*	7	LQH	0.5	0.1429	0.0040	0.9933
11	*Rhysida*	*aspinosa*	4	H	4.5	0.2500	0.0041	0.9802
12	*Rhysida*	*crassispina*	4	H	5.0	0.0000	0.0000	0.9705
13	*Rhysida*	*konda*	4	H	2.0	0.2500	0.1477	0.6317
14	*Rhysida*	*lewisi*	8	H	5.0	0.1250	0.0089	0.9638
15	*Rhysida*	*longipes*	9	H	1.5	0.3333	0.2284	0.6002
16	*Rhysida*	*pazhuthara*	5	L	3.5	0.2000	0.0020	0.9952
17	*Rhysida*	*sada*	6	Q	4.5	0.1667	0.0119	0.8331
18	*Rhysida*	sp. 1	3	H	5.0	0.0000	0.0000	0.7061
19	*Rhysida*	*trispinosa*	16	H	4.5	0.0000	0.1034	0.7059

### 3.2 Patterns of diversity and endemism

**FIGURE 2:**
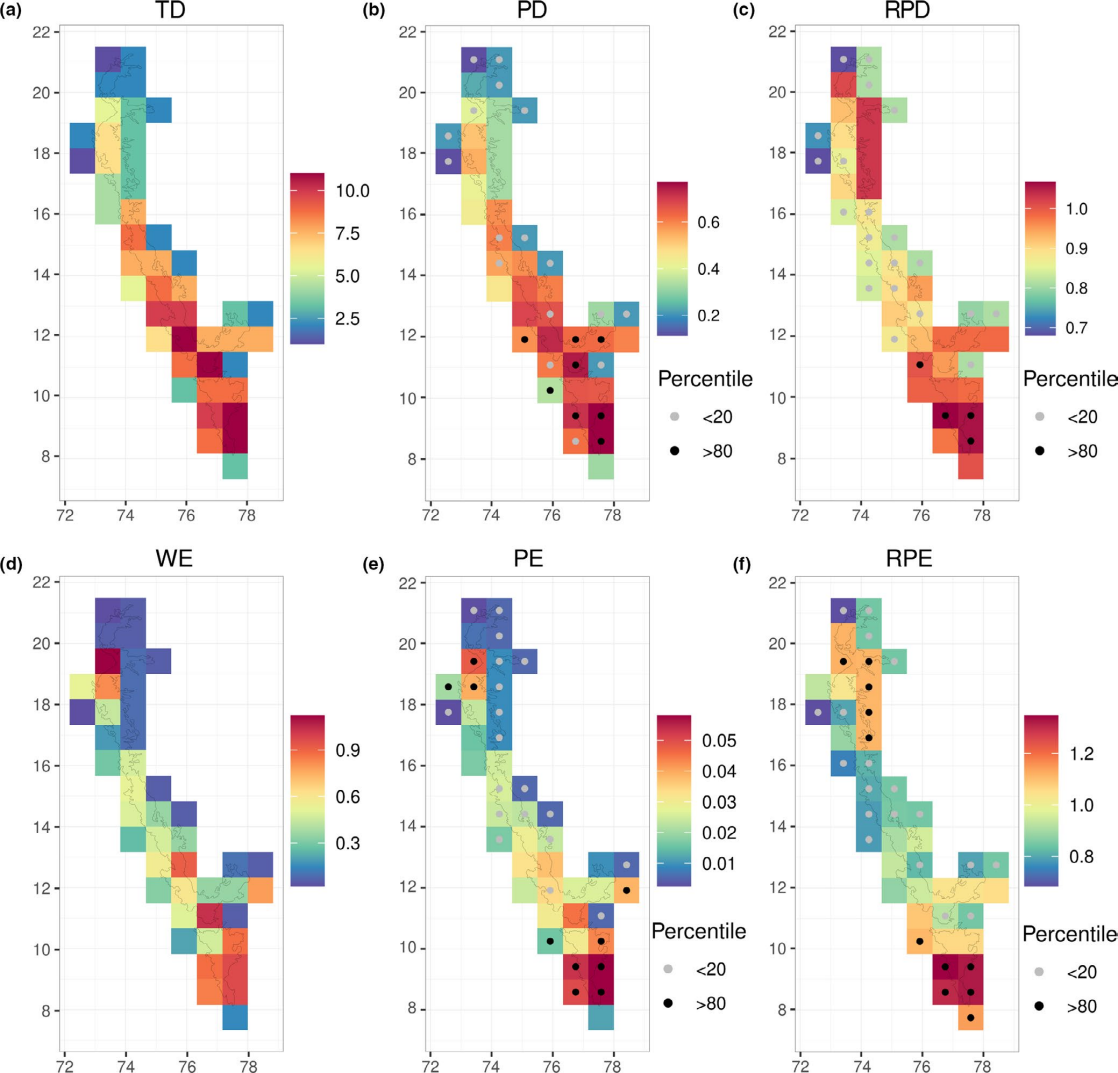
Maps of diversity and endemism indices for scolopendrid centipedes in the Western Ghats, India calculated at a resolution of 0.83° × 0.83° grid cells (a) taxonomic diversity presented as absolute counts of species in each grid cell, (b) phylogenetic diversity presented as sum of branch lengths (in millions of years) scaled by tree length, where large values correspond to greater phylogenetic diversity, (c) relative phylogenetic diversity, where values greater/lesser than 1 indicate greater relative proportion of older/younger lineages, (d) weighted endemism (taxonomic), where larger values correspond to greater species endemism, (e) phylogenetic endemism presented as proportion tree length, where large values correspond to greater phylogenetic endemism, and (f) relative phylogenetic endemism, where values greater/lesser than 1 indicate greater relative proportion of older/younger endemic lineages. For the phylogenetic indices, filled circles within cells indicate the percentile rank of the observed index within a null distribution of index values obtained by shuffling species occurrences across grid cells

The latitudinal trends in taxonomic and phylogenetic diversity were found to be broadly concordant within the WG (Figure 2a, 2b). The differences between these measures were mainly in the relative magnitude of diversity values. The southern WG had the highest values of phylogenetic diversity indices and also higher than expected values of phylogenetic diversity (Figure 2b) and relative phylogenetic diversity (Figure 2c) as compared to the null distribution. Higher than expected values of relative phylogenetic diversity indicate the presence of a greater proportion of lineages with long branch lengths. Nilgiris also had high values of taxonomic and phylogenetic diversity, but most values of relative phylogenetic diversity were not different from the null expectations (Figure 2b). Central WG followed next in the magnitude of phylogenetic diversity (Figure 2b) but had lower than expected values of relative phylogenetic diversity (Figure 2c), indicating the prevalence of species with short branch lengths. The northern WG had the lowest phylogenetic diversity (Figure 2b) but high values of relative phylogenetic diversity (Figure 2c).

When taxonomic and phylogenetic diversity were informed by species geographic ranges in the WG, regions in the southern WG converged in having both high and higher than expected values of weighted and phylogenetic endemism (Figure 2d, 2e). The southern WG was also associated with higher than expected values of relative phylogenetic endemism (Figure 2f), indicating that the species found here are characterized by small range sizes and a higher proportion of lineages with long branch lengths. The central WG had lower than expected values of both phylogenetic endemism and relative phylogenetic endemism, indicating a higher proportion of species with relatively wide distributions and short branch lengths. Most areas within the northern WG, including regions which varied in phylogenetic endemism, emerged as having higher than expected values of relative phylogenetic endemism (Figure 2f).

The patterns of diversity and endemism calculated using binary maps at the larger spatial scale broadly correspond to values derived from habitat suitability predictions in Maxent at the native resolution without applying a threshold (see Appendix S3), which is thought to more closely estimate these indices (Calabrese et al., 2014).

### 3.3 Patterns of species composition

**FIGURE 3:**
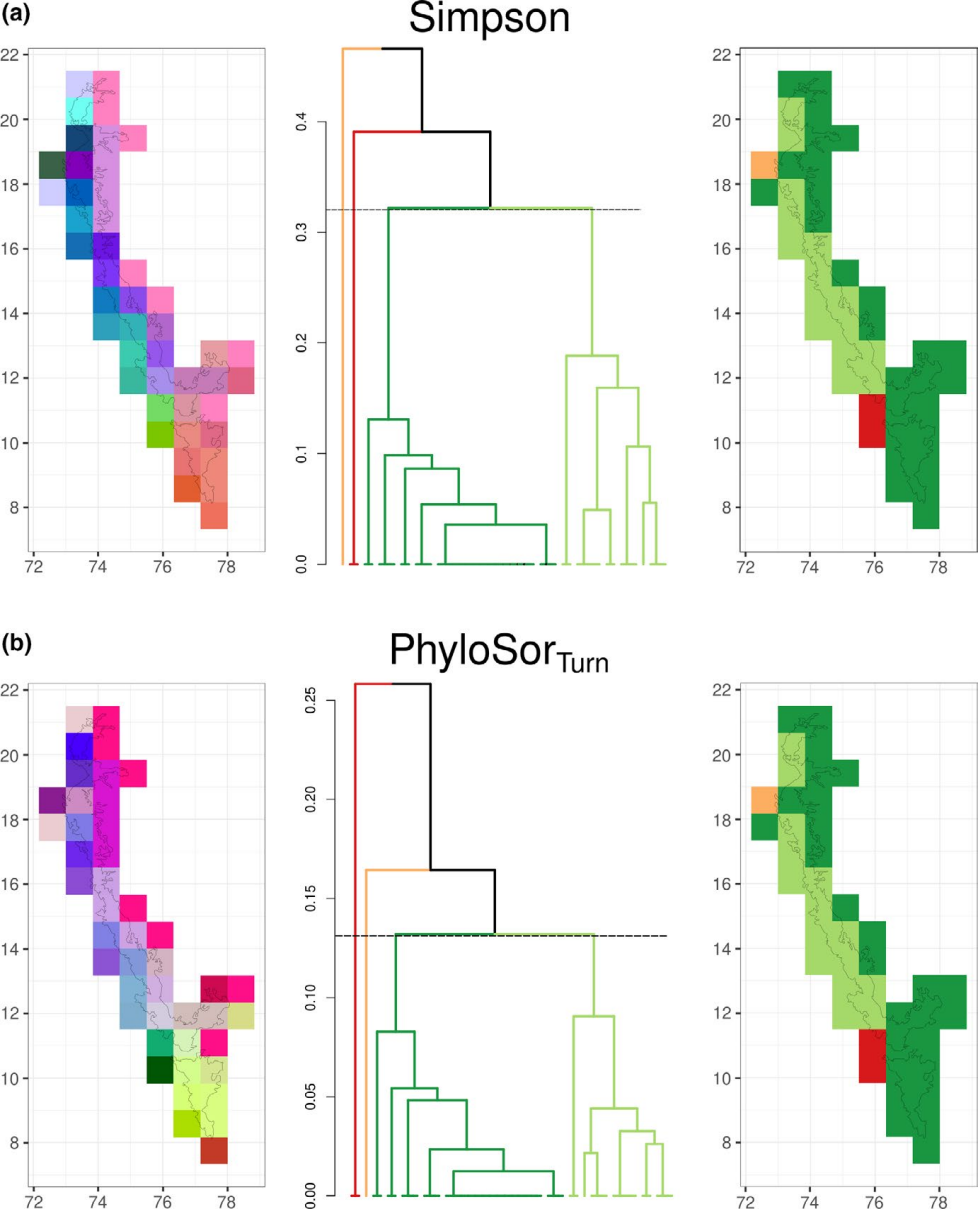
Patterns of (a) Simpson dissimilarity (species turnover) and (b) phylogenetic turnover (PhyloSor_Turn_) across the Western Ghats, India (maps in 0.83° × 0.83° grid cells). The left panel is compositional dissimilarity between grid cells represented as distance in color space where similar colors represent similar composition, and each plot needs to be viewed independently. Each plot was obtained by projecting the first three principal component axes of pairwise dissimilarity measures in the RGB scale. The center panel is a UPGMA tree generated from pairwise dissimilarity measures of composition, where the colors on the branches represent the different *k* = 4 clusters (dashed line representing the cutoff). The clusters recovered from the UPGMA tree are represented spatially in the panel on the right

Simpson dissimilarity and PhyloSor_Turn_, which describe the turnover component of taxonomic and phylogenetic beta diversity, indicated that grid cells within the southern WG and Nilgiris, along with a few cells on the eastern edge of the WG showed relatively greater compositional similarity. These cells differed in composition to most regions in the central and northern WG, which grouped separately. This was evident when the first three principal components of the dissimilarity matrix were plotted in RGB color space and also when thresholds were applied to the UPGMA tree of dissimilarity to retrieve four clusters (Figure 3a, 3b).

## 4 DISCUSSION

### 4.1 How is centipede diversity distributed in the WG?

This is one of the first studies to encompass the entire stretch of the Western Ghats (WG) to systematically evaluate the patterns of diversity, endemism, and composition using primary distribution data to model species distributions along with a robust, dated species‐level phylogeny. Measures of taxonomic and phylogenetic diversity show support for predictions from the latitudinal diversity gradient (LDG) and southern WG refuge hypotheses, where we expect centipede diversity to decrease from lower to higher latitudes in the WG.

The decline in diversity with increasing latitude is a well‐known pattern across taxa and regions (Hillebrand, 2004; ants: Economo et al., 2018; angiosperms: Kerkhoff et al., 2014; mammals: Rolland et al., 2014). Various explanations have been proposed to explain LDG, where higher species richness at lower latitudes has been attributed to greater time available for speciation, higher speciation rates, or lower extinction rates (Mittelbach et al., 2007). The causal drivers of variation in speciation and extinction rates can be related to geographic area, productivity, time, climatic stability, temperature, and biological interactions (Fine, 2015). The southern WG may have acted as a refuge during Cretaceous volcanism (ca 65 Ma), which is thought to be associated with widespread extinctions of plant and animal species in the northern WG (Joshi & Karanth, 2013 and references therein). This, along with climatic stability over long periods of time as inferred from past vegetation patterns (Divya et al., 2020; Prasad et al., 2009), could have led to the persistence and diversification of ancient centipede lineages in the southern WG. Our findings support the expectations of the southern WG refuge hypothesis, in that the southern WG has higher taxonomic and phylogenetic diversity represented by relatively older lineages as compared to the other WG subregions. The decline in diversity in the higher latitudes of the WG can be additionally related to an increase in temperature and precipitation seasonality, which is associated with the Miocene emergence of the Indian monsoon (Gunnell, 1997) and has been implicated in the decline in taxonomic (Page & Shanker, 2020) and phylogenetic diversity in plants (Bose et al., 2019).

### 4.2 Are there hotspots within the hotspot?

We find that weighted and phylogenetic endemism broadly converged in the WG, where regions of high endemism represented by relatively older lineages were detected in the southern WG. Interestingly, plateaus with stunted forests in the northern WG also showed the presence of unique endemic lineages.

#### 4.2.1 Southern Western Ghats—hotspot within a hotspot

The examination of relative phylogenetic diversity and endemism patterns allowed us to identify ancient, evolutionarily, and geographically unique lineages in the southern WG, which highlights this subregion as a hotspot of conservation importance within the larger WG biodiversity hotspot. While high taxonomic diversity in the southern WG has been documented in plants (Page & Shanker, 2020) and animals (Aravind & Gururaja, 2011; Aravind et al., 2005; Daniels, 1992), an understanding of both diversity and endemism patterns from a phylogenetic perspective remains limited (Divya, 2019). Our current results, using three genera within Otostigminae, align with the inferences drawn from the single genus *Digitipes* (Joshi & Karanth, 2013), where biogeographic analysis suggested high phylogenetic diversity and endemism in the southern WG.

#### 4.2.2 Less diverse but unique northern Western Ghats

The northern WG had the lowest phylogenetic diversity, but there was an increase in phylogenetic endemism from central WG to some cells in the northern WG. This is related to the presence of narrow range endemics such as *Rhysida crassispina, R. sada,* and *Ethmostigmus sahyadrensis* in this subregion. The centipede community found here also had significantly high relative phylogenetic endemism, which was smaller in magnitude than the southern WG, but indicates a greater representation of relatively older endemic lineages (around 30–65 my) as compared to the central WG. These species are found in unique habitats consisting of stunted evergreen forests and adjoining grasslands on lateritic plateaus in the northern WG. These plateaus are geologically unique and are made up of basalt from Deccan Trap lava flows, which have weathered into lateritic rock that has further undergone various levels of erosion (Watve, 2013). They show a diversity of unique seasonal microhabitats (Thorpe et al., 2018) and have a distinct vegetation consisting of several endemic herbaceous species that show adaptations to surviving in poor soil conditions (Joshi & Janarthanam, 2004; Lekhak & Yadav, 2012).

Unfortunately, the network of protected areas in the northern WG is not as extensive as in the southern WG, though they consist of areas identified to be of high conservation value (Das et al., 2006; Watve, 2013). The protected areas in the northern WG are small in size and consist of fragmented forests with high anthropogenic disturbance located in the vicinity of urban centers (Gadgil, 2011; Thorpe & Watve, 2015). Apart from centipedes, there have been records of other range‐restricted species on these plateaus across different taxa (snails: Aravind et al., 2005; plants: Lekhak & Yadav, 2012; Shigwan et al., 2020; amphibians: Katwate et al., 2013). The present study highlights the need to systematically understand the evolutionarily unique species found in these plateaus across different taxonomic groups and identify key areas of conservation importance.

### 4.3 What are the patterns of species turnover across the WG?

Taxonomic and phylogenetic turnover in centipedes revealed two major clusters, which were largely restricted to either the southern WG and Nilgiris, or to the central and northern WG, suggesting that there might be possible species replacement around the Palghat Gap. The Palghat Gap is a 30–km‐wide valley interrupting the WG mountain chain, which has been identified as an important dispersal barrier for plants and animals based on distribution patterns (Subramanyam & Nayar, 1974) and genetic analyses (e.g., Joshi & Karanth, 2013; Robin et al., 2015; Vidya et al., 2005). Species from the WG that spanned the Palghat Gap based on observed occurrence locations include *Digitipes barnabasi*, *D*. *coonoorensis*, *D*. *jonesii*, *D*. *nudus, E. coonooranus, Rhysida longipes,* and *R*. *trispinosa*. These species also spanned this potential barrier based on their predicted distributions. The occurrence locations of *Ethmostigmus agasthyamalaiensis* were absent north of the Palghat gap, but it was predicted to occur in some grid cells of the Nilgiris. All the remaining species were distributed either north or south of the Palghat Gap.

There is increasing evidence that biogeographic barriers in addition to the climatic barriers can shape community dynamics across tropical areas. In the tropical Andes, river valleys and elevation have been shown to drive distribution and phylogenetic breaks in endemic bird taxa. These barriers are found to encompass areas with high richness of narrowly distributed species (Hazzi et al., 2018). Major rivers also demarcate bioregions which explain distribution patterns of anurans in Amazonia, followed in importance by the climatic and topographic variation seen in this region (Godinho & da Silva, 2018). In the Australian monsoon tropics, biogeographic barriers have shaped the distribution patterns in plants and several animal taxa (Edwards et al., 2017). Our results recommend the simultaneous assessment of geoclimatic factors while examining patterns of diversity and endemism in the WG, given its complex geological past and the contemporary gradient in temperature and precipitation seasonality.

### 4.4 Centipede distributions within and outside the Western Ghats

The predictions of the species distribution models correspond to the known habitat affiliations of the centipede genera studied here, where *Digitipes* and *Ethmostigmus* are found in wet forests, while *Rhysida* species vary in their habitat requirements and are more widely distributed in peninsular India. For some species, predictions of habitat suitability extend into biogeographic regions where the species have not been currently reported but share similar habitats. For example, the range of *E*. *tristis* (an Eastern Ghats endemic) is predicted in a few cells of the southern WG and Nilgiris, and the predicted range of the widespread *R*. *trispinosa* extends into the eastern boundary of the WG. The presence of these species in their range extremes along with species having more extensive distributions across this mountain range may lead to unique centipede communities in the eastern edge of the WG.

The niche modeling predictions of relative habitat suitability can serve as a useful guide for sampling effort to assess population‐level patterns and processes in centipedes of the WG. The predicted distributions of many species extend beyond the latitudinal range of their observed presence locations, which needs to be investigated to confirm their range boundaries (see Appendix S2). These include distributions of species from the northern and central WG such as *E. conooranus*, *E*. *praveeni,* and *R*. *lewisi,* which are predicted to have distributions extending further north as compared to currently reported occurrences. *Ethmostigmus sahyadrensis* is a northern WG species, while *D*. *jangii* is also found in the central WG and both have more southern predicted distributions as compared to their observed occurrences. The models developed here can also be used to predict potential distributions in other undersampled areas such as wet forests of the Eastern Ghats and north‐east India.

### 4.5 Limitations of a presence‐background modeling approach

Maxent is a presence‐background approach for species distribution modeling that has been shown to perform consistently well across a range of sample sizes as compared to other model algorithms (Wisz et al., 2008). Studies have also shown that Maxent models can provide useful predictions with even 5–10 presence locations and that prediction accuracy improves for species with small range sizes related to strong environmental gradients (Hernandez et al., 2006). This modeling approach has also been shown to be less sensitive to prediction inaccuracies arising from predictor complexity (De Marco & Nóbrega, 2018) and tolerates correlations in predictor variables (Elith et al., 2011). However, Maxent lacks information on prevalence (proportion of presence locations in the model extent), which is necessary to calculate the conditional probability of presence given the environment at a location (Merow et al., 2013). In the absence of this information, Maxent provides predictions of habitat suitability that cannot be compared across models differing in their background. In addition to this, model evaluation is difficult when absence data are not available, as true absences and false presences in model predictions cannot be accurately estimated (Leroy et al., 2018). Despite these caveats, we believe that our use of model transferability measures to arrive at the optimal model and obtain habitat suitability predictions are helpful in surveying potential areas of distribution, which can be used to build a robust presence–absence dataset for future work.

To summarize, we demonstrated the use of primary distribution data along with species distribution modeling and a detailed species‐level phylogeny to understand diversity gradients and identify hotspots of endemism in an ancient soil arthropod group within tropical wet forests. We discuss our results in the light of past climatic stability, contemporary patterns in seasonality as well as geography—factors shown to influence diversity and distribution patterns in the WG and globally. Our results from soil arthropods highlight the need for macroecological analyses on a diverse range of taxa to understand diversity and endemism patterns and evaluate their generality in these diverse tropical forests. These would also allow us to compare the relative importance of geological processes and climatic variables in shaping these patterns across a spectrum of life‐history traits and evolutionary histories. This approach involving both ecological and evolutionary factors also promises to be useful in identifying areas of endemism across taxa within the biodiversity hotspot, an important exercise for identifying areas of conservation importance.

## CONFLICT OF INTEREST

The authors declare no conflict of interest.

## AUTHOR CONTRIBUTION

**D. K. Bharti:** Conceptualization (supporting); Formal analysis (equal); Methodology (equal); Visualization (lead); Writing‐original draft (equal). **Greg Edgecombe:** Data curation (supporting); Investigation (equal); Resources (equal); Writing‐review & editing (equal). **Praveen Karanth:** Data curation (supporting); Formal analysis (supporting); Investigation (equal); Resources (equal); Writing‐review & editing (equal). **Jahnavi Joshi:** Conceptualization (lead); Data curation (lead); Formal analysis (equal); Funding acquisition (lead); Methodology (equal); Visualization (supporting); Writing‐original draft (equal).

### OPEN RESEARCH BADGES

This article has been awarded Open Data Badge. All materials and data are publicly accessible via the Open Science Framework at https://github.com/bhartidk/centipede_diversity_endemism.

## Supporting information

Appendix S1

Appendix S2

Appendix S3

## Data Availability

The location data used in this study are provided in Appendix S1. The environmental layers used for species distribution modeling are available for download from https://www.worldclim.org/data/bioclim.html. R scripts used for building species distribution models, generating input files for Biodiverse 3.1, calculating indices, and generating plots are publicly available at https://github.com/bhartidk/centipede_diversity_endemism.
