# Supplementary material for: Spatial patterns of phylogenetic diversity and endemism in the Western Ghats, India: A case study using ancient predatory arthropods
Source: Ecol Evol. 2021 Nov 12;11(23):16499–513. doi: 10.1002/ece3.8119 (PMC8668739; doi:10.1002/ece3.8119)
Supplement: Supplementary file 2 — Appendix S2 [file ECE3-11-16499-s002.docx]

**Appendix S2.** Additional information on Maxent species distribution modeling approach and results.

**Sampling bias**

With unequal sampling effort in a complex landscape with strong latitudinal and elevation gradients, randomly picked background locations can lead to a poor estimate of environmental variation in the model extent, biasing predictions towards sampled environments rather than capturing species preferences (Phillips et al., 2009; Elith et al., 2011). To correct for this, we built a model of sampling bias with all surveyed locations and used its predictions (Fig. S2.1) to weigh the selection of background locations for species distribution models (following Phillips et al., 2009). The same set of background locations was used across all species being modelled.

**Predictor variables**

We used three sets of predictor variables – 1. all WorldClim variables (Fick & Hijmans, 2017) and soil type (ATREE Spatial Archive, 2020) (21 predictors), 2. preselected variables from WorldClim and soil type based on ecological intuition (7 predictors), and 3. primary variables from WorlClim and soil type layer (8 predictors) (Table S2.2). Maxent models were run using all three predictor datasets using the same procedure for feature class selection and model tuning as described in the main text. The WorldClim variables were used at 30 arcsecond resolution (~1 km at the equator), and are derived from monthly data on temperature, precipitation, solar radiation, wind speed and water vapour pressure, summarized over the years 1971-2000, and elevation was derived from SRTM data (http://srtm.csi.cgiar.org/).

The first predictor dataset uses all available predictor variables and lets the Maxent algorithm choose the ones that are most relevant in predicting habitat suitability. This approach can be useful when modeling multiple species, where there might be interspecific differences in relevant environmental variables and where their omission can lead to prediction error (Arau ́jo & Peterson, 2012). However, using all available predictors may lead to the inclusion of correlated variables, which increases model complexity, makes it difficult to tease apart the role of the causal predictor from its correlates and can lead to prediction error if the underlying correlation structure changes when extrapolating the model in space or time (Dormann et al., 2013). This can be especially problematic when small datasets are used for model training, where the resulting model is strongly influenced by a few points. The second predictor dataset offers a useful alternative when species biology is well known, allowing for the screening of predictors that are most relevant to the study species (Merow, Smith & Silander, 2013). However, insufficient knowledge of species biology or the need to model multiple species differing in their ecological requirements would require species-based specification of causal drivers, which may not always be possible. The third predictor dataset uses a wide breadth of environmental predictors but reduces their number by removing secondary variables (derived from the mean, minimum and maximum). Models using this reduced dataset have been shown to perform well in terms of transferability by avoiding overfitting to training data (Low et al., 2021).

**Model evaluation and selection**

We used two means of selecting the best performing model for each species among models built using different feature classes and tuning parameters – 1. based on model transferability using a cross-validation approach (omission rate, difference in test and training AUC – Area Under the receiver-operator Curve) and discriminatory power (AUC of test data) (Radosavljevic & Anderson, 2014) and 2. based on model parsimony (AICc – Akaike Information Criterion corrected for small smple sizes) (Warren & Seifert, 2011). The former helps in selecting models that do not overfit training data while predicting the test data well, and the latter measures the goodness-of-fit of a model while penalizing for the number of model parameters (Low et al., 2021). However, species distribution models selected using AICc may overpenalize parameters, especially when using hinge features, and may lead to underparametrized models which overpredict habitat suitability across the model extent (Low et al., 2021). We qualitatively inspected the continuous habitat suitability maps and thresholded binary maps to compare the predictions of models built using different combinations of the predictor dataset and the method of model selection. We found that models selected solely based on AICc overpredicted distribution for several species, while the three predictor datasets varied in their level of overprediction across species. Models built using ecologically preselected predictor variables seemed to overpredict more often than the other two predictor datasets. A summary of results for models built using the three predictor datasets and two different model selection methods is presented in Table S2.3.

**References**

Araújo, M. B., & Peterson, A. T. (2012). Uses and misuses of bioclimatic envelope modeling. *Ecology*, *93*(7), 1527–1539. https://doi.org/10.1890/11-1930.1

Dormann, C. F., Elith, J., Bacher, S., Buchmann, C., Carl, G., Carré, G., Marquéz, J. R. G., Gruber, B., Lafourcade, B., Leitão, P. J., Münkemüller, T., McClean, C., Osborne, P. E., Reineking, B., Schröder, B., Skidmore, A. K., Zurell, D., & Lautenbach, S. (2013). Collinearity: A review of methods to deal with it and a simulation study evaluating their performance. *Ecography*, *36*(1), 27–46. https://doi.org/10.1111/j.1600-0587.2012.07348.x

Elith, J., Phillips, S. J., Hastie, T., Dudík, M., Chee, Y. E., & Yates, C. J. (2011). A statistical explanation of MaxEnt for ecologists. *Diversity and Distributions*, *17*(1), 43–57. https://doi.org/10.1111/j.1472-4642.2010.00725.x

Fick, S. E., & Hijmans, R. J. (2017). WorldClim 2: New 1-km spatial resolution climate surfaces for global land areas. *International Journal of Climatology*, *37*(12), 4302–4315. https://doi.org/10.1002/joc.5086

Low, B. W., Zeng, Y., Tan, H. H., & Yeo, D. C. J. (2021). Predictor complexity and feature selection affect Maxent model transferability: Evidence from global freshwater invasive species. *Diversity and Distributions*, *27*(3), 497–511. https://doi.org/10.1111/ddi.13211

Merow, C., Smith, M. J., & Silander, J. A. (2013). A practical guide to MaxEnt for modeling species’ distributions: What it does, and why inputs and settings matter. *Ecography*, *36*(10), 1058–1069. https://doi.org/10.1111/j.1600-0587.2013.07872.x

Phillips, S. J., Dudík, M., Elith, J., Graham, C. H., Lehmann, A., Leathwick, J., & Ferrier, S. (2009). Sample selection bias and presence-only distribution models: Implications for background and pseudo-absence data. *Ecological Applications*, *19*(1), 181–197. https://doi.org/10.1890/07-2153.1

Radosavljevic, A., & Anderson, R. P. (2014). Making better Maxent models of species distributions: Complexity, overfitting and evaluation. *Journal of Biogeography*, *41*(4), 629–643. https://doi.org/10.1111/jbi.12227

Warren, D. L., & Seifert, S. N. (2011). Ecological niche modeling in Maxent: The importance of model complexity and the performance of model selection criteria. *Ecological Applications*, *21*(2), 335–342. https://doi.org/10.1890/10-1171.1

**Table S2.2.** Details of three different predictor datasets used for building Maxent models for each species.

| **Predictor dataset** | **Environmental variables** |
| --- | --- |
| 1. All WorldClim +Elevation + Soil type | 1. ann_mean_temp  2. mean_temp_warmQ  3. mean_temp_coldQ  4. ann_prec  5. prec_wet_month  6. prec_dry_month  7. prec_season  8. prec_wetQ  9. prec_dryQ  10. prec_warmQ  11. prec_coldQ  12. mean_diur_range  13. isotherm  14. temp_season  15. max_temp  16. min_temp  17. ann_range_temp  18. mean_temp_wetQ  19. mean_temp_dryQ  20. elevation  21. soil_stype |
| 2. Ecologically preselected variables | 1. ann_mean_temp  2. ann_prec  3. prec_season  4. prec_dryQ  5. min_temp  6. elevation  7. soil_stype |
| 3. Primary WorldClim + Elevation + Soil type | 1. ann_mean_temp  2. max_temp  3. min_temp  4. ann_prec  5. prec_wet_month  6. prec_dry_month  7. elevation  8. soil_stype |

**Variable codes:**

**ann_mean_temp –** annual mean temperature**, mean_temp_warmQ –** mean temperature of the warmest quarter, **mean_temp_coldQ** – mean temperature of the coldest quarter, **ann_prec** – annual precipitation, **prec_wet_month** – precipitation of the wettest month, **prec_dry_month** – precipitation of the driest month, **prec_season** – precipitation seasonality, **prec_wetQ** – precipitation of the wettest quarter, **prec_dryQ** – preciptation of the driest quarter, **prec_warmQ** – precipitation of the warmest quarter, **prec_coldQ** – precipitation of the coldest quarter, **mean_diur_range** – mean diurnal range of temperature, **isotherm –** isothermality, **temp_season** – temperature seasonality, **max_temp** – maximum temperature of the warmest month, **min_temp** – minimum temperature of the coldest month, **ann_range_temp** – annual range of temperature, **mean_temp_wetQ** – mean temperature of the wettest quarter, **mean_temp_dryQ** – mean temperature of the driest quarter, **soil_stype** – soil sub-type (includes 18 categories within the model extent including – 1. Calciorthids; 2. Haplagolls; 3. Haplaquents; 4. Haplustalfs, Palestalfs, Rhodustalf; 5. Natrargids, Salargids, Salorthids; 6. Ochraquats, Rhodustults, Haplustult; 7. Paleustalfs, Haplaquents; 8. Pelluderts, Pellusterts, Chromuster; 9. Pellusterts, Chromusters; 10. Plinhaqualts, Plinthustults, Plinth; 11. Quartzipsamments ; 12. Rhodustalfs, Pellusterts; 13. Tropoqualfs; 14. Troposaprists; 15. Udifluvents; 16. Ustipsamments; 17. Ustochrepts; 18. Water Body).

-

| Genus | Species | n | Predictor dataset | Model selection method | FC | RM | OR_MTP_ | AUC_DIFF_ | AUC_TEST_ | delta AICc |
| --- | --- | --- | --- | --- | --- | --- | --- | --- | --- | --- |
| *Digitipes* | *barnabasi* | 23 | all | Transferability | L | 5 | 0.0400 | 0.0100 | 0.6700 | 15.1300 |
|  |  |  |  | Parsimony | Q | 4 | 0.0833 | 0.0404 | 0.7458 | 0.0000 |
|  |  |  | ecology | Transferability | L | 5 | 0.0417 | 0.0117 | 0.6118 | 25.7494 |
|  |  |  |  | Parsimony | Q | 2 | 0.1667 | 0.1268 | 0.7313 | 0.0000 |
|  |  |  | primary | Transferability | L | 5 | 0.0417 | 0.0117 | 0.6118 | 22.9150 |
|  |  |  |  | Parsimony | Q | 0.5 | 0.1667 | 0.1531 | 0.6960 | 0.0000 |
| *Digitipes* | *coonoorensis* | 14 | all | Transferability | LQ | 0.5 | 0.0714 | 0.0415 | 0.9400 | 44.7731 |
|  |  |  |  | Parsimony | LQ | 2 | 0.1429 | 0.0337 | 0.9400 | 0.0000 |
|  |  |  | ecology | Transferability | LQ | 0.5 | 0.0714 | 0.0381 | 0.9400 | 0.8298 |
|  |  |  |  | Parsimony | LQ | 1 | 0.0714 | 0.0440 | 0.9300 | 0.0000 |
|  |  |  | primary | Transferability | Q | 2 | 0.0714 | 0.0315 | 0.9452 | 2.8629 |
|  |  |  |  | Parsimony | LQ | 1 | 0.0714 | 0.0369 | 0.9406 | 0.0000 |
| *Digitipes* | *jangii* | 9 | all | Transferability | QH | 4.5 | 0.1111 | 0.0544 | 0.8788 | 42.2759 |
|  |  |  |  | Parsimony | L | 1 | 0.1111 | 0.0876 | 0.8710 | 0.0000 |
|  |  |  | ecology | Transferability | QH | 5 | 0.1111 | 0.0527 | 0.8379 | 26.6885 |
|  |  |  |  | Parsimony | LQ | 1 | 0.1111 | 0.0884 | 0.8638 | 0.0000 |
|  |  |  | primary | Transferability | Q | 4 | 0.1111 | 0.0602 | 0.9004 | 41.3523 |
|  |  |  |  | Parsimony | Q | 1 | 0.1111 | 0.0709 | 0.8855 | 0.0000 |
| *Digitipes* | *jonesii* | 27 | all | Transferability | H | 1 | 0.0417 | 0.0445 | 0.9147 | NA |
|  |  |  |  | Parsimony | QH | 3 | 0.2262 | 0.0713 | 0.8953 | 0.0000 |
|  |  |  | ecology | Transferability | H | 5 | 0.1429 | 0.0429 | 0.9054 | 57.4808 |
|  |  |  |  | Parsimony | Q | 0.5 | 0.2262 | 0.1491 | 0.8220 | 0.0000 |
|  |  |  | primary | Transferability | H | 1 | 0.0833 | 0.0617 | 0.8967 | 147.0043 |
|  |  |  |  | Parsimony | Q | 0.5 | 0.2262 | 0.1149 | 0.8527 | 0.0000 |
| *Digitipes* | *nudus* | 6 | all | Transferability | L | 2.5 | 0.1667 | 0.0542 | 0.9148 | 10.6307 |
|  |  |  |  | Parsimony | Q | 1 | 0.1667 | 0.1500 | 0.8195 | 0.0000 |
|  |  |  | ecology | Transferability | Q | 1 | 0.1667 | 0.0339 | 0.9335 | 2.3812 |
|  |  |  |  | Parsimony | Q | 1.5 | 0.1667 | 0.0364 | 0.9320 | 0.0000 |
|  |  |  | primary | Transferability | Q | 1 | 0.1667 | 0.0362 | 0.9325 | 0.0000 |
|  |  |  |  | Parsimony | Q | 1 | 0.1667 | 0.0362 | 0.9325 | 0.0000 |
| *Ethmostigmus* | *agasthyamalaiensis* | 6 | all | Transferability | H | 5 | 0.1667 | 0.0302 | 0.9475 | 12.8549 |
|  |  |  |  | Parsimony | L | 5 | 0.1667 | 0.0534 | 0.9153 | 0.0000 |
|  |  |  | ecology | Transferability | H | 5 | 0.1667 | 0.0287 | 0.9508 | 12.3783 |
|  |  |  |  | Parsimony | L | 2.5 | 0.1667 | 0.0770 | 0.9036 | 0.0000 |
|  |  |  | primary | Transferability | H | 5 | 0.1667 | 0.0302 | 0.9483 | 14.0045 |
|  |  |  |  | Parsimony | H | 2 | 0.1667 | 0.0481 | 0.9364 | 0.0000 |
| *Ethmostigmus* | *coonooranus* | 6 | all | Transferability | L | 5 | 0.1667 | 0.0270 | 0.7856 | 3.1736 |
|  |  |  |  | Parsimony | Q | 4 | 0.1667 | 0.0432 | 0.8499 | 0.0000 |
|  |  |  | ecology | Transferability | H | 3.5 | 0.1667 | 0.0562 | 0.7462 | 5.7201 |
|  |  |  |  | Parsimony | Q | 2 | 0.1667 | 0.1123 | 0.7235 | 0.0000 |
|  |  |  | primary | Transferability | L | 3 | 0.1667 | 0.0484 | 0.8063 | 18.7029 |
|  |  |  |  | Parsimony | Q | 1.5 | 0.1667 | 0.0635 | 0.8974 | 0.0000 |
| *Ethmostigmus* | *praveeni* | 4 | all | Transferability | L | 1 | 0.2500 | 0.0069 | 0.9874 | NA |
|  |  |  |  | Parsimony | H | 4.5 | 0.2500 | 0.0118 | 0.9644 | 0.0000 |
|  |  |  | ecology | Transferability | Q | 0.5 | 0.2500 | 0.0160 | 0.9760 | NA |
|  |  |  |  | Parsimony | L | 2.5 | 0.2500 | 0.0344 | 0.9134 | 0.0000 |
|  |  |  | primary | Transferability | LQ | 0.5 | 0.2500 | 0.0199 | 0.9726 | 0.0000 |
|  |  |  |  | Parsimony | L | 3.5 | 0.2500 | 0.0318 | 0.9578 | 0.0000 |
| *Ethmostigmus* | *sahyadrensis* | 5 | all | Transferability | H | 1.5 | 0.2000 | 0.0058 | 0.9900 | NA |
|  |  |  |  | Parsimony | NA | NA | NA | NA | NA | NA |
|  |  |  | ecology | Transferability | H | 1 | 0.2000 | 0.0053 | 0.9909 | NA |
|  |  |  |  | Parsimony | Q | 2.5 | 0.2000 | 0.0087 | 0.9861 | 0.0000 |
|  |  |  | primary | Transferability | LQH | 0.5 | 0.2000 | 0.0041 | 0.9930 | 0.0000 |
|  |  |  |  | Parsimony | H | 3.5 | 0.2000 | 0.0093 | 0.9803 | 0.0000 |
| *Ethmostigmus* | *tristis* | 7 | all | Transferability | LQ | 4.5 | 0.1429 | 0.0032 | 0.9750 | 35.7237 |
|  |  |  |  | Parsimony | L | 3.5 | 0.1429 | 0.0069 | 0.9617 | 0.0000 |
|  |  |  | ecology | Transferability | H | 5 | 0.0000 | 0.0000 | 0.7061 | 15.0815 |
|  |  |  |  | Parsimony | L | 2.5 | 0.1429 | 0.0070 | 0.9572 | 0.0000 |
|  |  |  | primary | Transferability | LQH | 0.5 | 0.1429 | 0.0040 | 0.9933 | 0.0000 |
|  |  |  |  | Parsimony | LQH | 2.5 | 0.1429 | 0.0065 | 0.9872 | 0.0000 |
| *Rhysida* | *aspinosa* | 4 | all | Transferability | H | 4.5 | 0.2500 | 0.0041 | 0.9802 | 15.9678 |
|  |  |  |  | Parsimony | Q | 2 | 0.5000 | 0.0597 | 0.9269 | 0.0000 |
|  |  |  | ecology | Transferability | H | 2.5 | 0.2500 | 0.0095 | 0.9779 | NA |
|  |  |  |  | Parsimony | LQ | 2 | 1.0000 | 0.0346 | 0.9509 | 0.0000 |
|  |  |  | primary | Transferability | H | 4.5 | 0.2500 | 0.0041 | 0.9802 | 18.3761 |
|  |  |  |  | Parsimony | H | 1.5 | 0.2500 | 0.0101 | 0.9751 | 0.0000 |
| *Rhysida* | *crassispina* | 4 | all | Transferability | H | 5 | 0.0000 | 0.0000 | 0.9705 | NA |
|  |  |  |  | Parsimony | NA | NA | NA | NA | NA | NA |
|  |  |  | ecology | Transferability | H | 5 | 0.0000 | 0.0000 | 0.9705 | NA |
|  |  |  |  | Parsimony | H | 2 | 0.2500 | 0.0001 | 0.9998 | 0.0000 |
|  |  |  | primary | Transferability | H | 5 | 0.0000 | 0.0000 | 0.9705 | 40.5516 |
|  |  |  |  | Parsimony | Q | 4.5 | 0.2500 | 0.0001 | 0.9997 | 0.0000 |
| *Rhysida* | *konda* | 4 | all | Transferability | H | 3 | 0.2500 | 0.0280 | 0.9436 | NA |
|  |  |  |  | Parsimony | H | 5 | 0.0000 | 0.0000 | 0.5000 | 0.0000 |
|  |  |  | ecology | Transferability | H | 1 | 0.5000 | 0.1852 | 0.7910 | NA |
|  |  |  |  | Parsimony | L | 5 | 0.2500 | 0.0315 | 0.4911 | 0.0000 |
|  |  |  | primary | Transferability | H | 2 | 0.2500 | 0.1477 | 0.6317 | 1.8101 |
|  |  |  |  | Parsimony | H | 5 | 0.0000 | 0.0000 | 0.5000 | 0.0000 |
| *Rhysida* | *lewisi* | 8 | all | Transferability | H | 5 | 0.1250 | 0.0089 | 0.9638 | 13.3619 |
|  |  |  |  | Parsimony | H | 3 | 0.1250 | 0.0218 | 0.9577 | 0.0000 |
|  |  |  | ecology | Transferability | L | 0.5 | 0.1250 | 0.0183 | 0.9637 | 0.0000 |
|  |  |  |  | Parsimony | L | 0.5 | 0.1250 | 0.0183 | 0.9637 | 0.0000 |
|  |  |  | primary | Transferability | H | 5 | 0.1250 | 0.0089 | 0.9638 | 34.0498 |
|  |  |  |  | Parsimony | LQ | 0.5 | 0.2500 | 0.0194 | 0.9642 | 0.0000 |
| *Rhysida* | *longipes* | 9 | all | Transferability | NA | NA | NA | NA | NA | NA |
|  |  |  |  | Parsimony | L | 5 | 0.2222 | 0.1283 | 0.4064 | 0.0000 |
|  |  |  | ecology | Transferability | NA | NA | NA | NA | NA | NA |
|  |  |  |  | Parsimony | L | 5 | 0.1111 | 0.0652 | 0.4064 | 0.0000 |
|  |  |  | primary | Transferability | H | 1.5 | 0.3333 | 0.2284 | 0.6002 | 0.0000 |
|  |  |  |  | Parsimony | L | 5 | 0.0000 | 0.0000 | 0.5000 | 0.0000 |
| *Rhysida* | *pazhuthara* | 5 | all | Transferability | H | 2 | 0.2000 | 0.0023 | 0.9942 | NA |
|  |  |  |  | Parsimony | L | 5 | 0.4000 | 0.0040 | 0.9919 | 0 |
|  |  |  | ecology | Transferability | L | 0.5 | 0.2000 | 0.0020 | 0.9932 | 18.83 |
|  |  |  |  | Parsimony | LQ | 2 | 0.2000 | 0.0026 | 0.9925 | 0 |
|  |  |  | primary | Transferability | L | 3.5 | 0.2000 | 0.0020 | 0.9952 | 25.0622 |
|  |  |  |  | Parsimony | H | 3 | 0.2000 | 0.0024 | 0.9941 | 0.0000 |
| *Rhysida* | *sada* | 6 | all | Transferability | LQH | 2.5 | 0.1667 | 0.0030 | 0.9919 | NA |
|  |  |  |  | Parsimony | Q | 4.5 | 0.1667 | 0.0121 | 0.9235 | 0.0000 |
|  |  |  | ecology | Transferability | LQ | 0.5 | 0.1667 | 0.0131 | 0.9705 | NA |
|  |  |  |  | Parsimony | L | 2.5 | 0.1667 | 0.0144 | 0.9170 | 0.0000 |
|  |  |  | primary | Transferability | Q | 4.5 | 0.1667 | 0.0119 | 0.8331 | 24.4715 |
|  |  |  |  | Parsimony | H | 2 | 0.1667 | 0.0303 | 0.9094 | 0.0000 |
| *Rhysida* | sp. 1 | 3 | all | Transferability | H | 5 | 0.0000 | 0.0000 | 0.7061 | 0.0000 |
|  |  |  |  | Parsimony | L | 5 | 1.0000 | 0.4720 | 0.3083 | 0.0000 |
|  |  |  | ecology | Transferability | H | 5 | 0.0000 | 0.0000 | 0.7061 | 2.7802 |
|  |  |  |  | Parsimony | H | 2 | 0.0000 | 0.0000 | 0.7061 | 0.0000 |
|  |  |  | primary | Transferability | H | 5 | 0.0000 | 0.0000 | 0.7061 | 2.7802 |
|  |  |  |  | Parsimony | L | 1.5 | 0.6667 | 0.2800 | 0.5417 | 0.0000 |
| *Rhysida* | *trispinosa* | 16 | all | Transferability | H | 5 | 0.0000 | 0.1153 | 0.6948 | 0.4947 |
|  |  |  |  | Parsimony | H | 4.5 | 0.0000 | 0.1155 | 0.6934 | 0.0000 |
|  |  |  | ecology | Transferability | H | 3 | 0.0625 | 0.1087 | 0.6473 | 41.7734 |
|  |  |  |  | Parsimony | L | 5 | 0.0625 | 0.0534 | 0.4703 | 0.0000 |
|  |  |  | primary | Transferability | H | 4.5 | 0.0000 | 0.1034 | 0.7059 | 1.2883 |
|  |  |  |  | Parsimony | L | 5 | 0.1250 | 0.0920 | 0.4501 | 0.0000 |


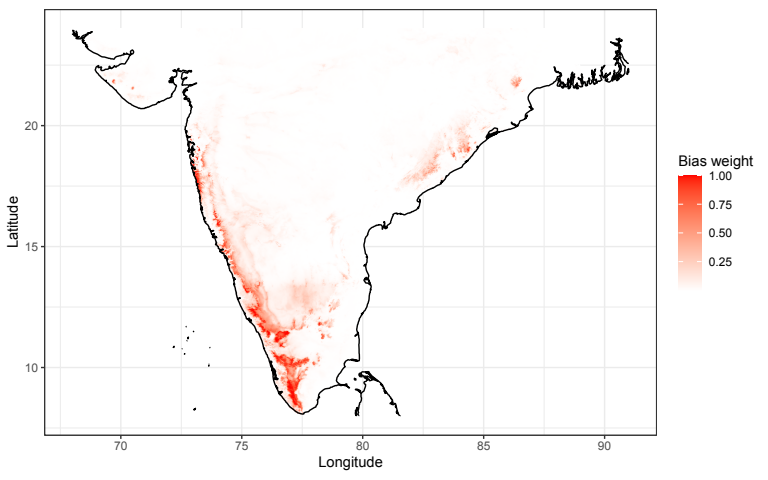


**Figure S2.1.** Prediction map of the sampling bias model which was used to weigh selection of background locations for the Maxent species distribution models.


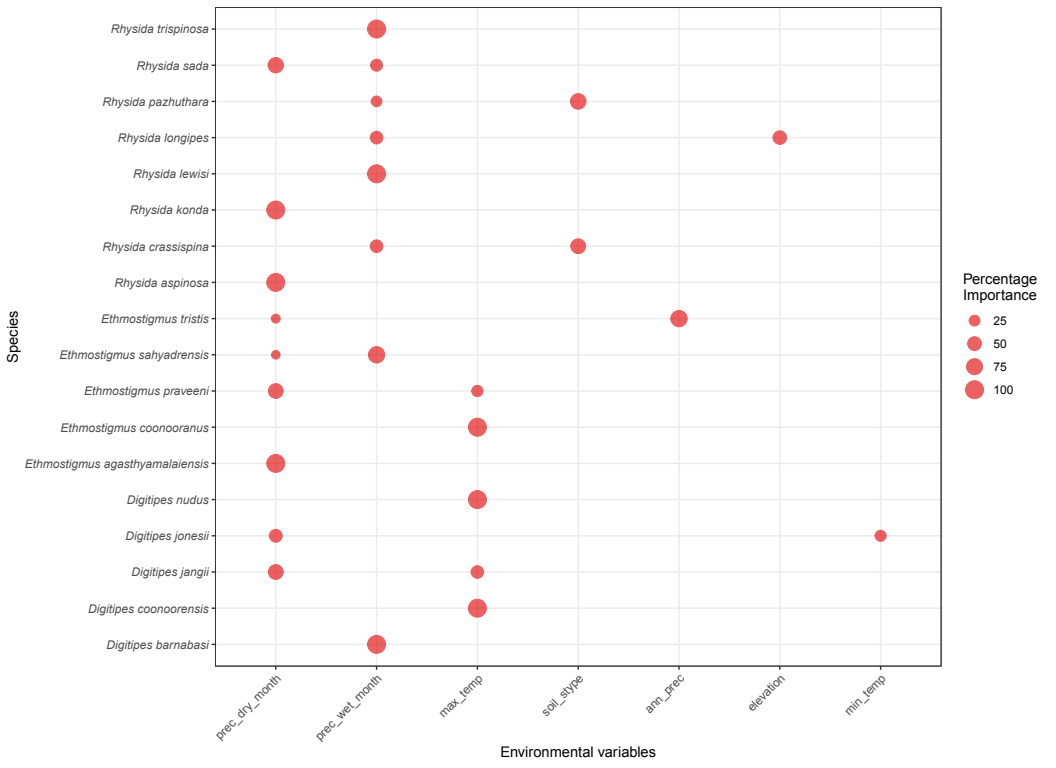


**Figure S2.2.** Permutation importance of variables used to run Maxent species distribution models for each species. Variables with the highest two values of permutation importance are indicated using the position of the filled circle on the x-axis, while the size of the circle corresponds to the magnitude of percentage permutation importance.

**Variable codes:**

**prec_dry_month** – precipitation of the driest month, **prec_wet_month** – precipitation of the wettest month, **max_temp** – maximum temperature of the warmest month, **soil_stype** – soil subtype, **ann_prec** – annual precipitation, **min_temp** – minimum temperature of the coldest month


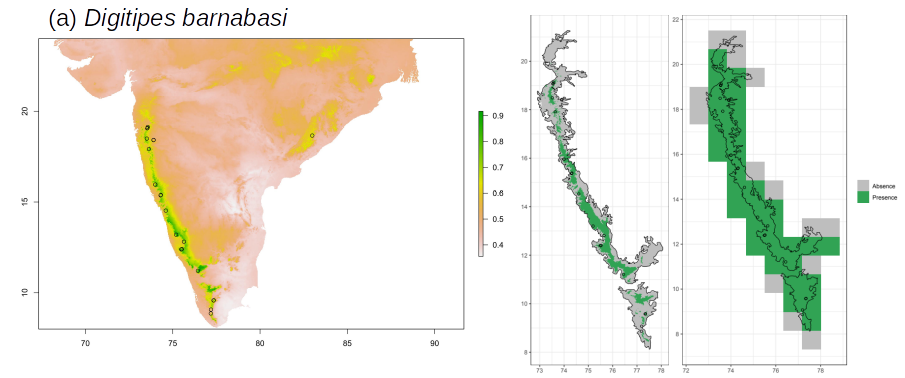


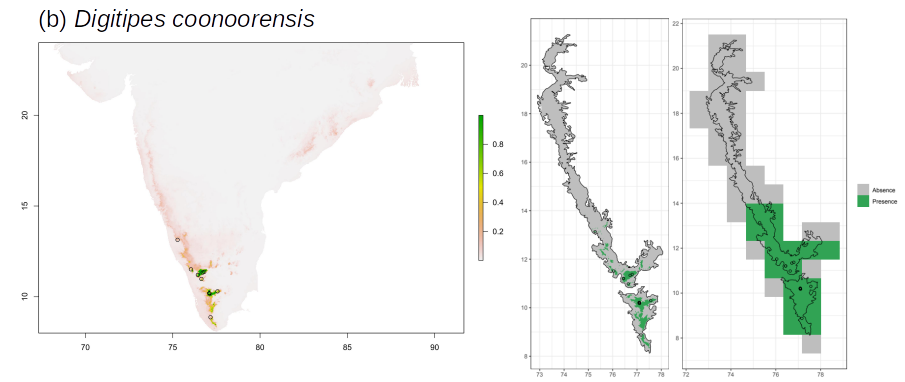


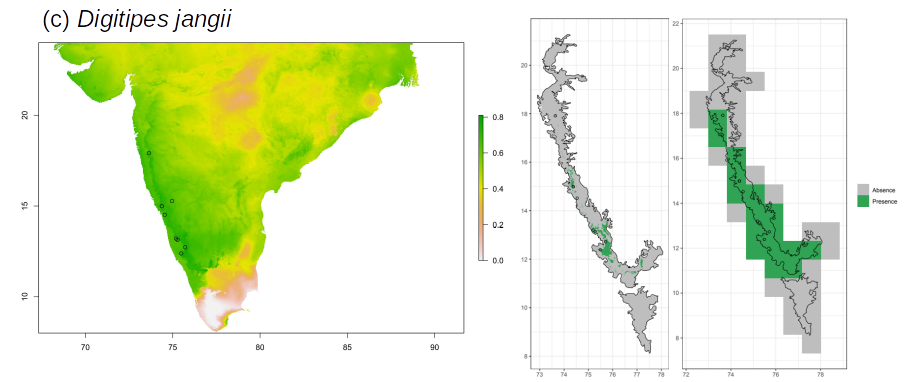


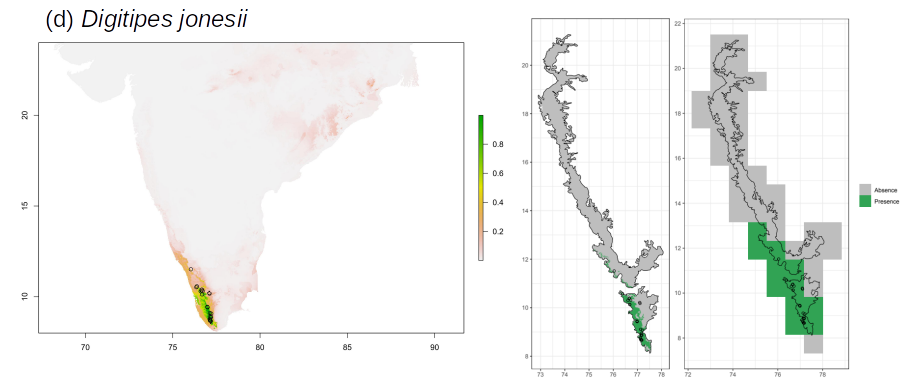


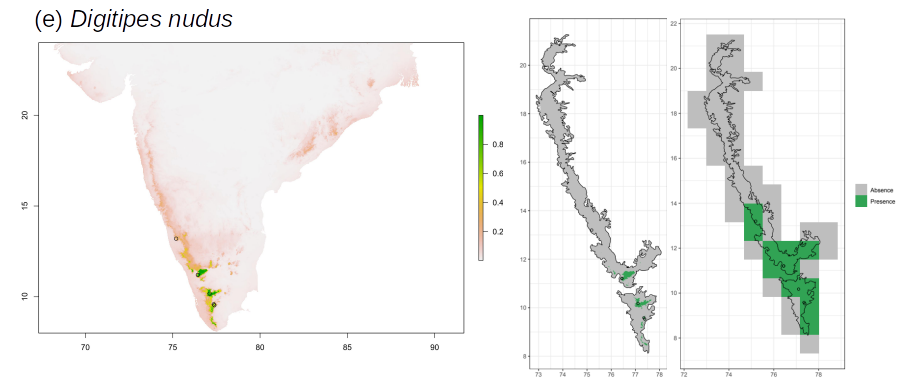

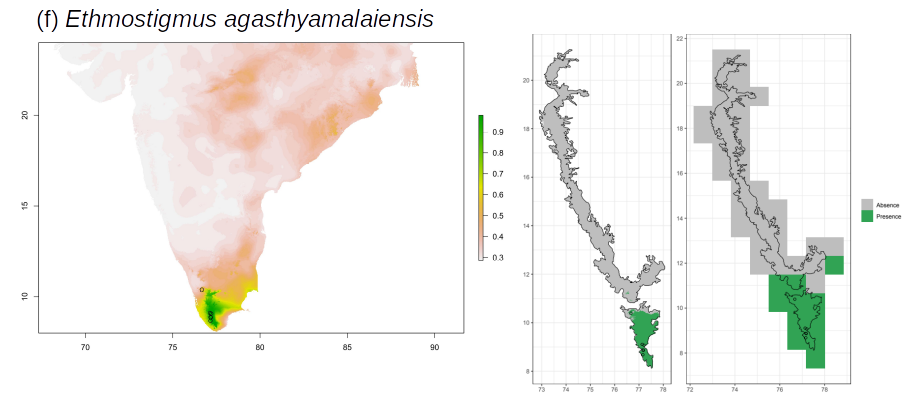


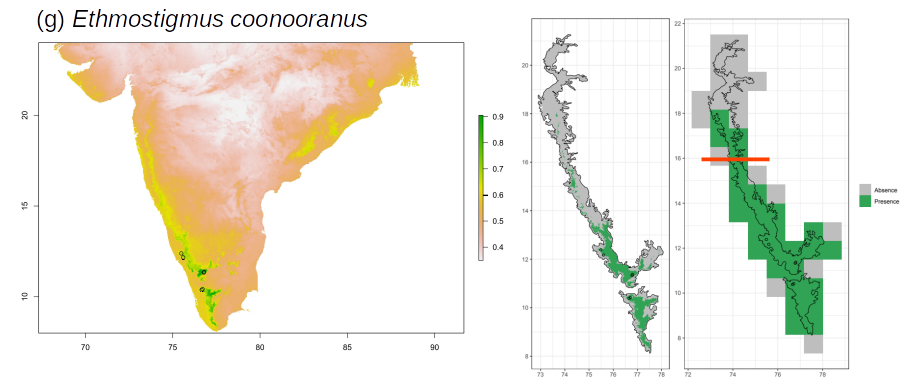


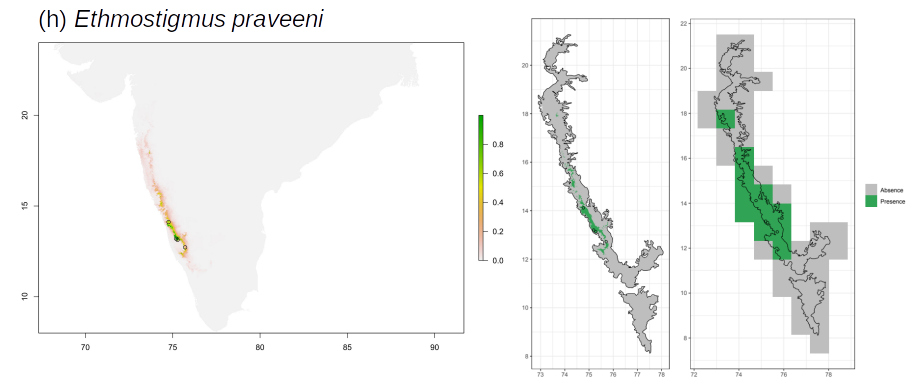


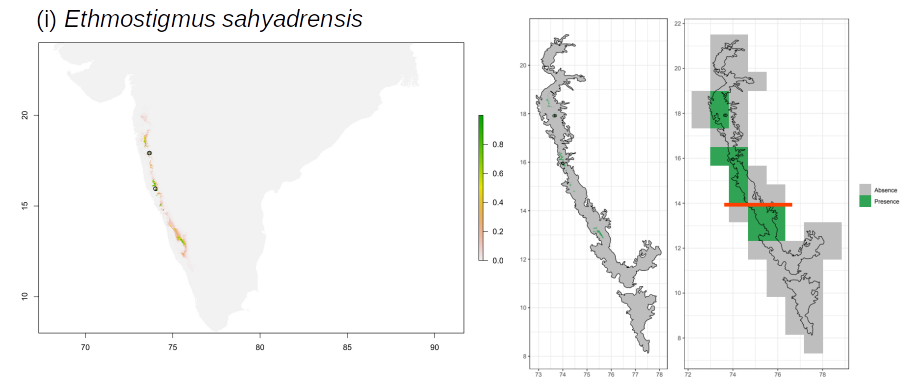


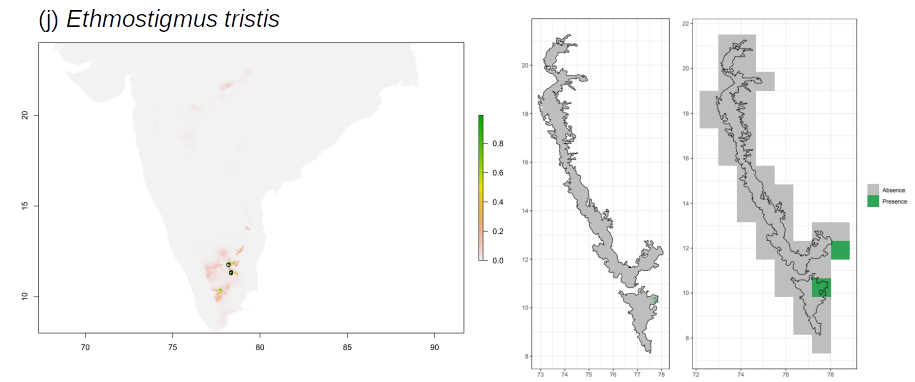


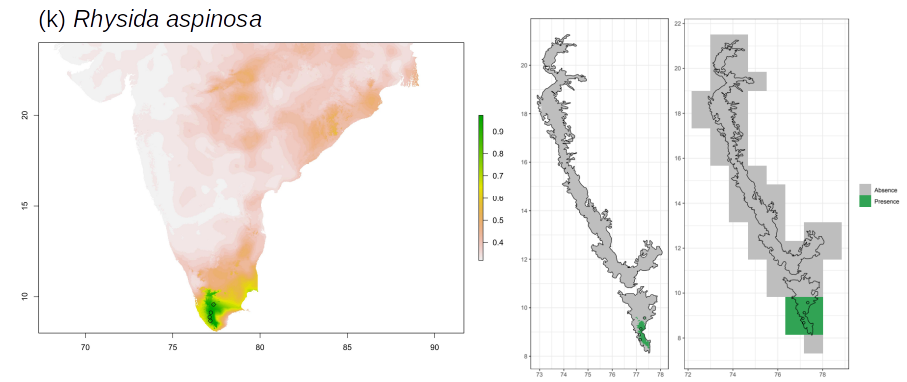


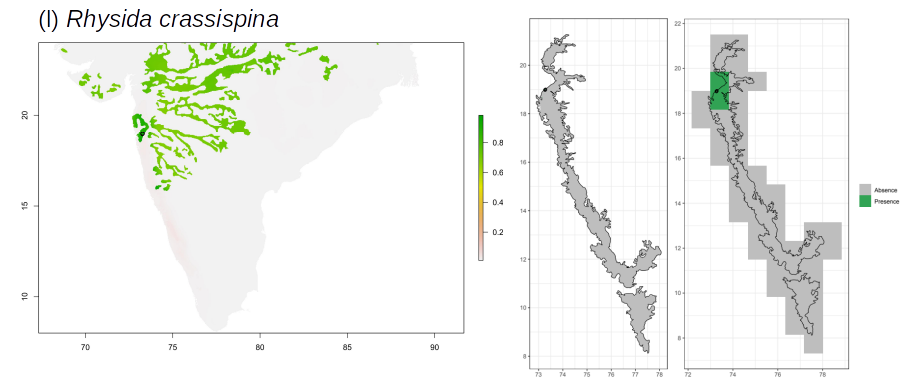


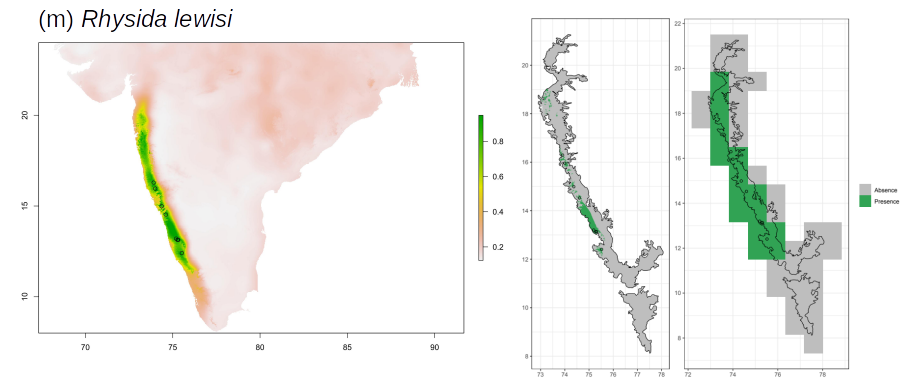


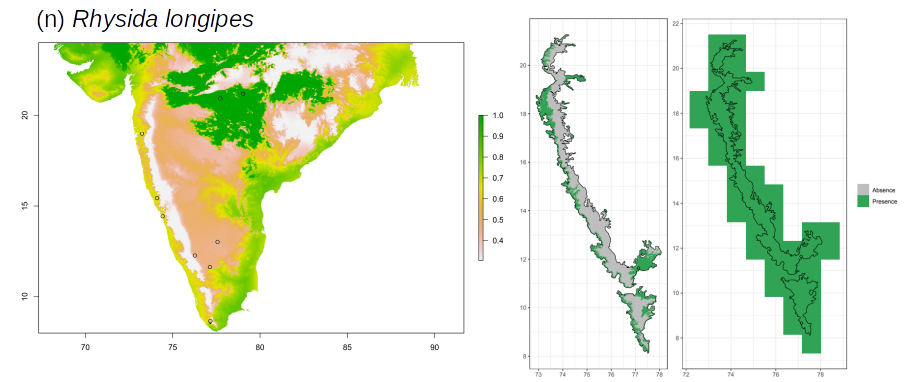


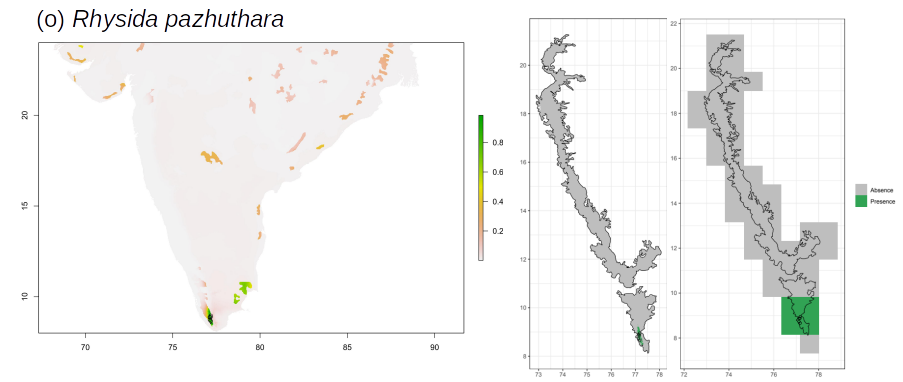


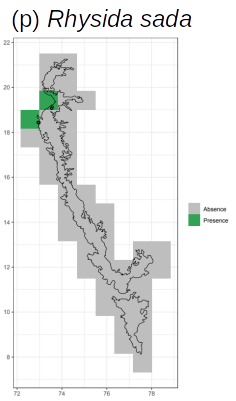

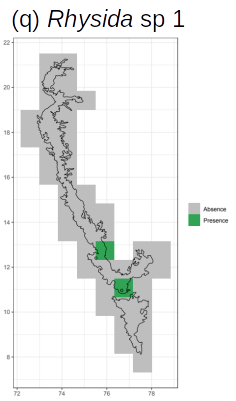


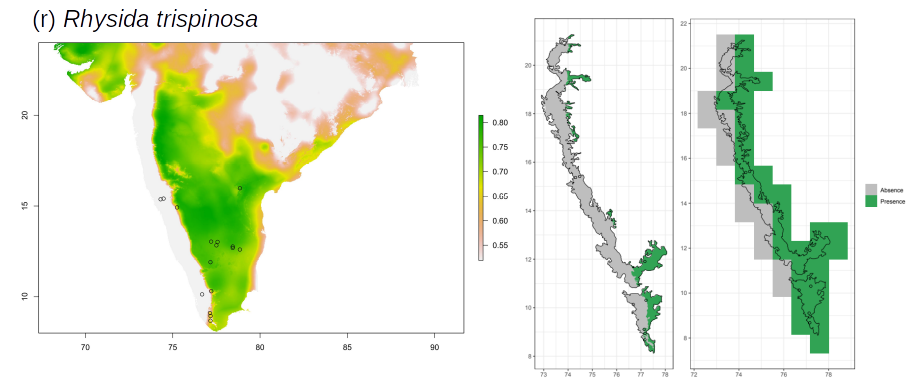


**Figure S2.3.** For each species, Maxent predictions are presented as continuous habitat suitability maps for peninsular India at 0.083° ✕ 0.083° resolution from Maxent (left), presence-absence map for the Western Ghats at 0.083° ✕ 0.083° resolution obtained after applying a threshold of maximum sum of sensitivity and specificity and retaining cells above this value (center) and presence-absence map for the Western Ghats aggregated to 0.83° ✕ 0.83° resolution (right).

For the two species listed below, the red bars in the sub-figure on the right panel represent limits applied to the predicted range to remove small pockets of predicted habitat suitability located substantially farther away from observed presence locations: *Ethmostigmus coonooranus –* removed predictions >16°N, *E. sahyadrensis –* removed predictions <14°N
