# Supplementary material for: Spatial patterns of phylogenetic diversity and endemism in the Western Ghats, India: A case study using ancient predatory arthropods
Source: Ecol Evol. 2021 Nov 12;11(23):16499–513. doi: 10.1002/ece3.8119 (PMC8668739; doi:10.1002/ece3.8119)
Supplement: Supplementary file 3 — Appendix S3 [file ECE3-11-16499-s003.docx]

**Appendix S3.** Diversity and endemism measures calculated using continuous predictions of habitat suitability from Maxent species distribution models.


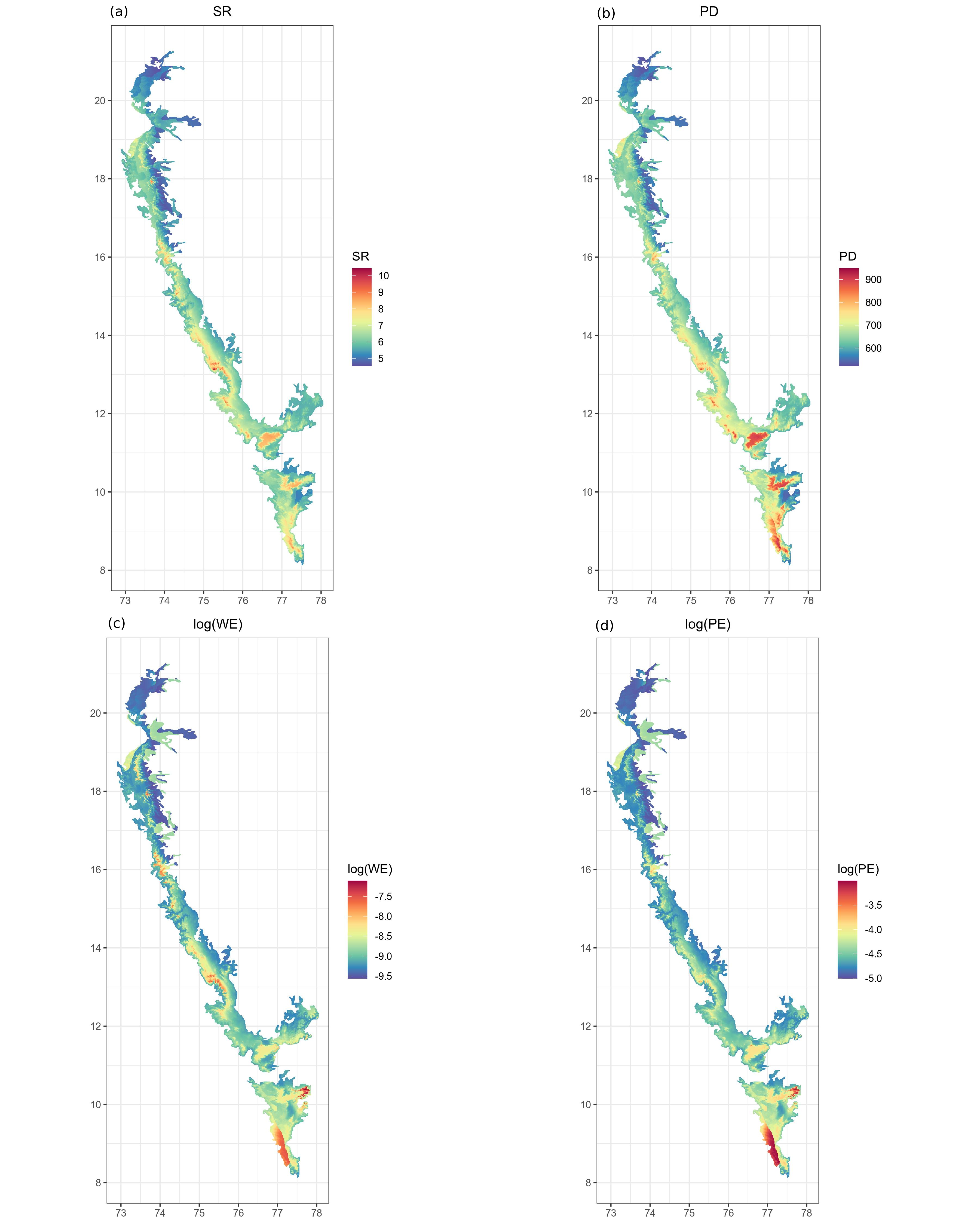
**Figure S3.4.** Maps of diversity indices for scolopendrid centipedes, (A) species richness (taxonomic), (B) phylogenetic diversity (sum of branch lengths), (C) natural log of weighted endemism, (D) natural log of phylogenetic endemism, in the Western Ghats, India in 0.083° ✕ 0.083° grid cells.
